# A papain-like cysteine protease-released small signal peptide confers wheat resistance to wheat yellow mosaic virus

**DOI:** 10.1038/s41467-023-43643-y

**Published:** 2023-11-27

**Authors:** Peng Liu, Chaonan Shi, Shuang Liu, Jiajia Lei, Qisen Lu, Haichao Hu, Yan Ren, Ning Zhang, Congwei Sun, Lu Chen, Yaoyao Jiang, Lixiao Feng, Tianye Zhang, Kaili Zhong, Jiaqian Liu, Juan Zhang, Zhuo Zhang, Bingjian Sun, Jianping Chen, Yimiao Tang, Feng Chen, Jian Yang

**Affiliations:** 1 State Key Laboratory for Managing Biotic and Chemical Threats to the Quality and Safety of Agro-products, Key Laboratory of Biotechnology in Plant Protection of Ministry of Agriculture and Rural Affairs and Zhejiang Province, Institute of Plant Virology, Ningbo University, Ningbo, 315211 China; 2 National Key Laboratory of Wheat and Maize Crop Science/CIMMYT-China Wheat and Maize Joint Research Center/Agronomy College, Henan Agricultural University, Zhengzhou, 450002 China; 3 Hunan Plant Protection Institute, Hunan Academy of Agricultural Sciences, Changsha, 410152 China; 4 Institute of Hybrid Wheat, Beijing Academy of Agriculture and Forestry Sciences, Beijing, 100097 China

**Keywords:** Plant immunity, Agricultural genetics, Biotic, Viral pathogenesis

## Abstract

Wheat yellow mosaic virus (WYMV), a soil-borne pathogen, poses a serious threat to global wheat production. Here, we identify a WYMV resistance gene, *TaRD21A*, that belongs to the papain-like cysteine protease family. Through genetic manipulation of *TaRD21A* expression, we establish its positive role in the regulation of wheat to WYMV resistance. Furthermore, our investigation shows that the TaRD21A-mediated plant antiviral response relies on the release of a small peptide catalyzed by TaRD21A protease activity. To counteract wheat resistance, WYMV-encoded nuclear inclusion protease-a (NIa) suppress TaRD21A activity to promote virus infection. In resistant cultivars, a natural variant of TaRD21A features a alanine to serine substitution and this substitution enables the phosphorylation of Serine, thereby weakening the interaction between NIa and TaRD21A, reinforcing wheat resistance against WYMV. Our study not only unveils a WYMV resistance gene but also offers insights into the intricate mechanisms underpinning resistance against WYMV.

## Abstract (web summary)

Soil-borne wheat yellow mosaic virus (WYMV) poses a serious threat to global wheat production. Here, the authors report that the nuclear inclusion protease-a produced by WYMV interacts with a small peptide catalyzed by TaRD21A protease activity to mediate WYMV resistance through activating MAPK signaling pathway.

## Introduction

Wheat yellow mosaic virus (WYMV), a significant pathogen affecting wheat (*Triticum aestivum*) across East Asia, induces pronounced yellow mosaic symptoms in infected plants, causing yield reductions of up to 70%^1,2^. WYMV is prevalent in many wheat-growing regions along the Yangtze River in China^1^ and belongs to a member of the genus *Bymovirus* in the *Potyviridae* family. Its genome consists of two segments. WYMV RNA1 spans approximately 7.6 kb and encodes a sizable polyprotein (~270 kDa), subsequently cleaved into eight mature proteins through protease action. WYMV RNA2 measures around 3.5 kb and encodes a polyprotein (~100 kDa), further processed into two proteins: a 28 kDa P1 protein and a 73 kDa P2^3^. Nuclear inclusion protease-a (NIa), among the viral proteins, shapes amorphous nuclear inclusions upon cell infection, displaying serine protease activity^4^. WYMV infection relies on intricate interactions between WYMV-encoded proteins and host factors^5,6^. Furthermore, these viral proteins can also engage with cellular elements, leading to the suppression or circumvention of the host multilayered antiviral responses^6,7^.

WYMV is transmitted by the root-infecting parasite *Polymyxa graminis*^1,8^. Because the dormant spores of *P. graminis* are thick-walled and can survive for a long time, it is difficult to eliminate WYMV inside the dormant spores of *P. graminis* via conventional field management or pesticide applications^8^. Therefore, cultivation of wheat varieties resistant to WYMV is the most effective way to control the virus in practice. To date, several genes or quantitative trait loci (QTLs) that provide resistance against WYMV infection have been identified on chromosomes 2 A, 2DL, 3BS and 5AL^9^. Among these genes, *YmYF* (527.4–629.6 Mb), *YmIb* (582.5–728.6 Mb), *Q.Ymym* (582.5–629.6 Mb) and *Qym1* (577.4–629.6 Mb) are located on chromosome 2DL and appear to be at the same locus based on the physical position of the linked markers^10^, indicating that this locus on chromosome 2D is important for wheat resistance to WYMV infection. Recently, a resistance gene *Ym2* was isolated using positive cloning approach, which acts within the root either by hindering the initial movement of WYMV from the vector into the root and/or by suppressing viral multiplication^11^. However, the genetic and molecular mechanisms underlying wheat resistance to WYMV remain poorly understood.

Increasing evidence has demonstrated that papain-like cysteine proteases (PLCPs) represent the central hubs of plant immunity^12^. The roles of PLCPs in plant immunity are often verified experimentally by eliminating specific protease genes through gene knockout or RNA silencing (via RNA interference, RNAi). For example, compared with normal *Arabidopsis* plants, *Arabidopsis* plants with a defective PLCP RD21 protein are more susceptible to the necrotrophic fungal pathogen *Botrytis cinerea*^13^, and knocking down of the expression level of *Nicotiana benthamiana C14* gene resulted in an increased susceptibility to *Phytophthora infestans* infection^14,15^. Similarly, tomato *rcr3* null mutants are more susceptible to the fungus *Cladosporium fulvum* and the nematode *Globodera rostochiensis*^16,17^. In addition, PLCPs can release small peptides, known as damage-associated molecular patterns (DAMPs), to activate plant immunity. In *Zea mays*, for example, an immune signaling peptide released from a propeptide via PLCP activity activates salicylic acid signaling^18^. On the other hand, many members of different PLCP subfamilies can be targeted by a variety of unrelated pathogen effectors. For instance, the activities of maize CP1A, CP1B, XCP2, and CP2 can be inhibited by the Pit2 effector of *Ustilago maydis*^19^. The *P. infestans* effector AvrBlb2 has been shown to interact with tomato C14 to prevent its secretion into the apoplast^15^. Recent reports by Bar-Ziv and others have also indicated that during tomato yellow leaf curl virus (TYLCV) infection in plants, the viral V2 protein inhibits the activity of the host papain-like cysteine protease CYP1^20,21^. However, the mechanisms through which PLCPs activate plant antiviral immunity and through which viral proteins suppress PLCP-mediated host immune responses are still largely unknown.

In this work, we identify a WYMV resistance gene *TaRD21A* through genome wide association study (GWAS) and quantitative trait locus (QTL) mapping. We subsequently verify that TaRD21A positively modulates WYMV resistance by genetic transformation. We further find that a small peptide, released in the presence of TaRD21A from a propeptide, activates MAPK signaling, and thus is involved in the process of antiviral infection of plants. The WYMV-encoded NIa protein interacts with TaRD21A to prevent the protease activity of TaRD21A and promote WYMV infection. Moreover, we reveal a natural allele of *TaRD21A* in resistant wheat cultivars, and the protein encoded by this allele is phosphorylated at Ser-96, which weakens the interaction between TaRD21A and NIa. Our results provide insights into the arms race between wheat and WYMV.

## Results

### Discovery of a WYMV resistance candidate gene

**Fig. 1:**
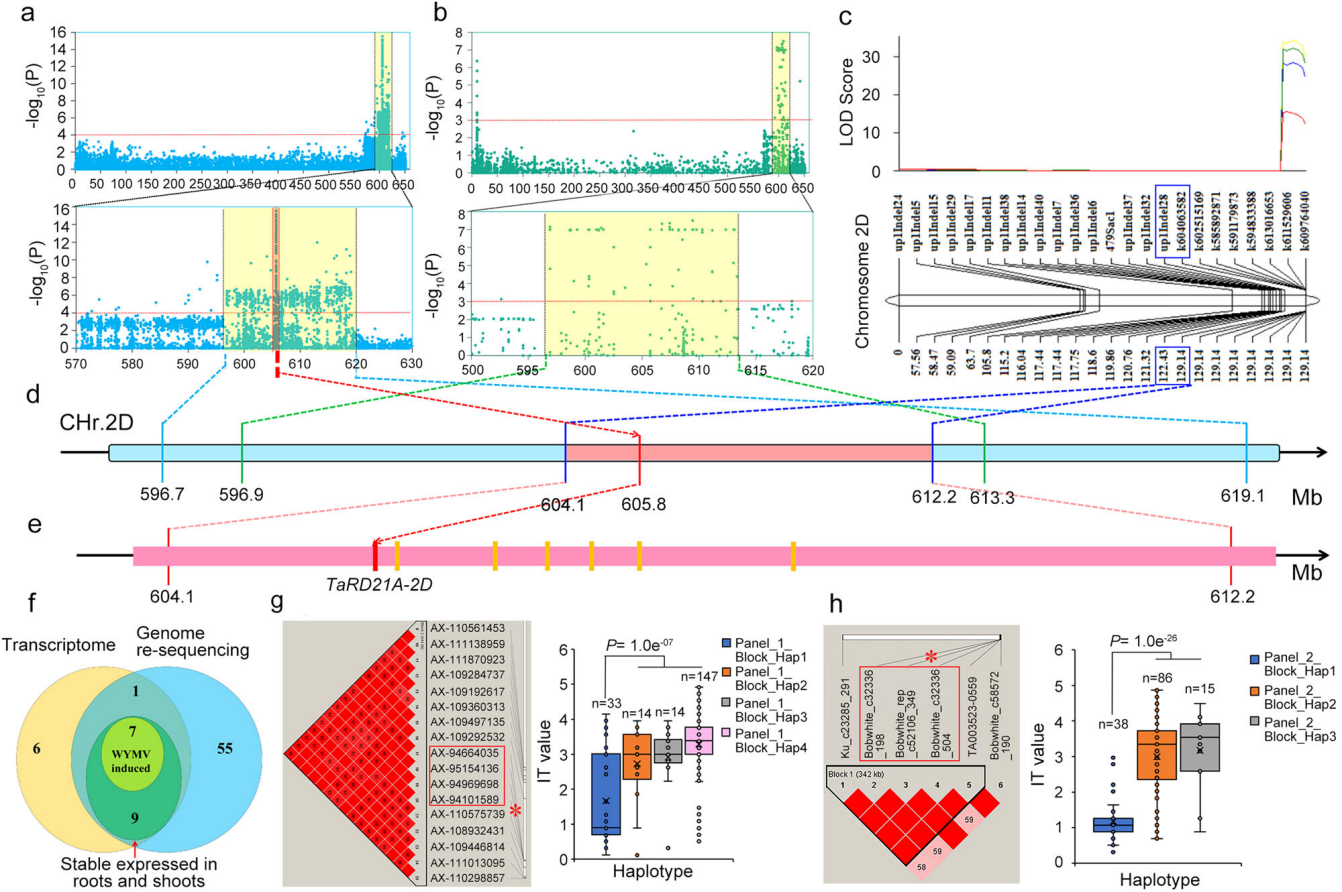
Identification of a candidate gene for wheat yellow mosaic virus (WYMV) resistance based on multiple strategies. **a**, **b** GWAS result for WYMV resistance on chromosome 2D in panel I (**a**) and panel II (**b**). The red horizontal line indicates the genome-wide significance threshold (*P* = 1.0e^−^^04^ in panel I and *P* = 1.0e^−03^ in panel II). Yellow background area indicates the candidate region (595.1-619.1 Mb in panel I and 596.9-613.3 Mb in panel II) on 2D. Two sides and the multiple test correction is conducted with Bonferroni method. **c** LOD contours for quantitative trait loci (QTL) to WYMV resistance on 2D identified by inclusive composite interval mapping (ICIM) in the UC1110/PI610750 (UP) population; **d** Integrated analysis of GWAS and QTL results. **e** Distribution of seven candidate genes in the region of 604.1–612.2 Mb. **f** Seven candidate genes were screened by multi-omics. Transcriptomics identified 23 differentially expressed genes (DEGs) in the target region by bulked segregant transcriptome using RNA-seq (BSR-Seq). Re-sequencing of the genome identified 17 of 23 DEGs with nonsynonymous variations or promoter variations in the target region between UC1110 and PI610750. The expression pattern identified 16 of 17 genes with a stable expression in roots and shoots in the target region, and 7 of 16 candidate genes were induced by WYMV infection. **g**, **h** Haplotype analysis indicated a 444-kb block covering the lead SNP (AX-95101589) in Panel I and a 342-kb block covering the lead SNP (BobWhite_c32336_504) in Panel II. For box-plot, the horizontal lines from top to bottom represent the maximum, first quartile, median, third quartile, and minimum of the total data, respectively. The cross in the middle of the box represents the average. *n* represents the number of wheat accessions with the corresponding haplotype. Statistics: for both datasets, two-sided *t* test was performed. *n* the number of wheat accessions with the corresponding haplotype. Source data are provided as a Source Data file.

Over a three-year period, the assessment of WYMV resistance traits in 406 tested wheat accessions revealed that 292 of them (71.9%) exhibited Infection Type (IT) values greater than 2 (Supplementary Fig. 1a). In our GWAS findings, we identified 1,173 significant SNPs, predominantly on chromosomes 2 A (146), 2B (138), and 2D (716) in Panel I, and 134 significant SNPs, primarily on chromosomes 2 A (19), 2B (19), and 2D (81) in Panel II (Supplementary Fig. 1b, Supplementary Data 1). Haplotype analysis of these significant tagSNPs revealed strong linkage disequilibrium (LD) in regions 596.7–619.1 Mb in Panel I and 596.9–613.3 Mb in Panel II on chromosome 2D (Fig. 1a-b and Supplementary Fig. 1c, d). These results collectively suggest the presence of a crucial WYMV resistance locus on chromosome 2D. Furthermore, we identified 136 significant SNPs on chromosome 2 A within the region of 731.1–750.2 Mb in Panel I and 18 significant SNPs in the region of 732.5-743.4 Mb in Panel II (Supplementary Fig. 1b, Supplementary Data 1). Similarly, on chromosome 2B, we found 128 significant SNPs in the region of 725.6–755.0 Mb in Panel I and 18 significant SNPs in the region of 725.9–742.5 Mb in Panel II (Supplementary Fig. 1b, Supplementary Data 1). Collinearity analysis indicated a strong collinearity between the three candidate regions on chromosomes 2 A, 2B, and 2D (Supplementary Fig. 1e). To further investigate these findings, we created two populations, UC1110/PI610750 (UP-RIL) and Bainong 64/Jingshuang 16 (BJ-DH), for linkage mapping. Bulk segregant analysis (BSA) results showed that different SNPs were mainly clustered on chromosome 2D in the regions of 588.8–614.6 Mb in UP-RIL (BSA-UP) and 573.9-613.1 Mb in BJ-DH (Supplementary Fig. 2a, b, Supplementary Data 2, 3). Linkage analysis unveiled a QTL, Qupwym.henau-2DL, with phenotypic variance explained (PVE) ranging from 13.7% to 39.9% in UP-RIL and a QTL, *Qbjwym.hau*−2DL, with PVE ranging from 20.4% to 31.4% in BJ-DH (Supplementary Data 4). Subsequently, we developed 27 markers targeting the 570-620 Mb region on chromosome 2D for fine mapping, guided by BSA and genome resequencing results of UC1110 and PI610750. In this process, we refined the location of *Qupwym.hau*−2DL to an interval between the k604063582 and up1Indel28 loci (604.1-612.2 Mb) (Fig. 1c, Supplementary Data 4). These results established that the *Qupwym.hau*−2DL locus spanned the region of 604.1–612.2 Mb, which encompassed 100 high-confidence genes (Fig. 1d, e). Further analysis utilizing BSR-seq revealed that 23 out of the 100 genes exhibited differential expression levels following WYMV infection between susceptible and resistant pools in the UP-RIL population (Fig. 1f, Supplementary Data 5). Genome resequencing of the two parental strains of UP-RIL showed that 21 genes exhibited polymorphisms between the two parents, with 17 of them displaying nonsynonymous variations or polymorphisms within their promoter regions (Fig. 1f, Supplementary Data 6). Given the progression of WYMV infection from roots to shoots in wheat plants, we selected 16 genes with elevated expression levels in roots and shoots for further analysis (Fig. 1f). qRT-PCR results confirmed that 7 of these 16 genes were induced after WYMV infection (Fig. 1f, Supplementary Fig. 2c). Upon closer examination, one candidate gene, *TraesCS2D02G513600*, was found to be located within a 444 kb block containing the lead SNP (AX-95101589) in Panel I (see Fig. 1g) and a 342 kb block containing the lead SNP (BobWhite_c32336_504) in Panel II (Fig. 1h). Additionally, rerunning linkage mapping using the developed markers in the BJ-DH population revealed that *Qbjwym.henau-*2DL was situated between up1Indel38 and up1Indel (600.0–605.8 Mb) (Supplementary Fig. 2d, Supplementary Data 4), and *TraesCS2D02G513600* was the sole candidate gene within the *Qbjwym.henau*−2DL region. Consequently, *TraesCS2D02G513600* emerged as the final candidate gene for WYMV resistance.

### TraesCS2D02G513600 is a papain-like cysteine protease (PLCP)

**Fig. 2:**
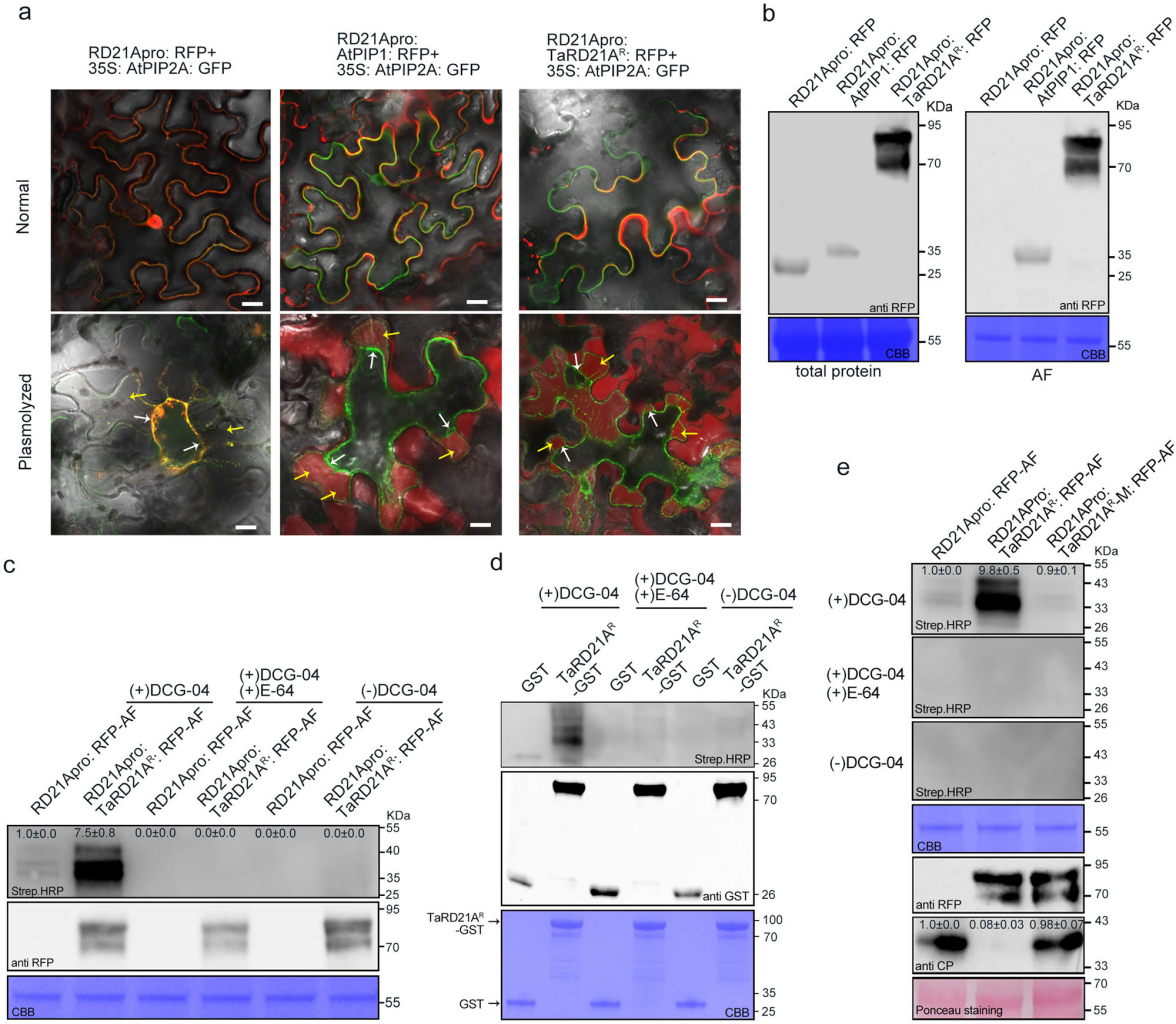
TaRD21A is a member of the papain-like cysteine proteases (PLCPs) family. **a** Subcellular localization of TaRD21A^R^ in *N. benthamiana* leaves. RFP under TaRD21A^R^ native promoter was used as negative control. RD21Apro: AtPIP1: RFP, RD21Apro: TaRD21A^R^: RFP or RD21Apro: RFP was co-expressed with plasma membrane (PM) marker 35 S: AtPIP2A: RFP in *N. benthamiana* leaves. Yellow arrows indicate apoplastic spaces and white arrows indicate PM. Bar, 10 µm. Three times each experiment was repeated independently with similar results. **b** The AF and total protein of the *N. benthamiana* leaves transiently expressing RD21Apro: AtPIP1: RFP, RD21Apro: TaRD21A^R^: RFP or RD21Apro: RFP were analyzed by western blot assay using an anti-RFP antibody. **c** AF were prepared from *N. benthamiana* leaves transiently expressing RD21Apro: TaRD21A^R^: RFP or RD21Apro: RFP and then labeled with DCG-04 in the presence or absence of E-64. **d** TaRD21A^R^-GST was expressed and purified from *E. coli* (BL21) and then co-incubated with DCG-04. The purified GST was used as control. **e** TaRD21A protease activity is involved in WYMV resistance in *N. benthamiana*. The conserved catalytic triad Cys-His-Asn in TaRD21A^R^ was substituted with Glycine (TaRD21A^R^-M) and then fused to C-terminus of RFP. AF from these assayed leaves was extracted for analysis protease activity. (−)DCG-04 in (**c**–**e**) indicated that the AF or GST fusion protein without DCG-04 labelling and used for distinguish from background signals. Coomassie Blue staining in (**b**–**e**) shows the assayed protein loaded. The data in (**b**–**e**) are representative of *n* = 3 independent experiments. Source data are provided as a Source Data file.

Our *in-silico* sequence analysis revealed that the TraesCS2D02G513600 protein consists of 463 amino acids (aa) and features a cathepsin propeptide inhibitor domain, a peptidase_C1 domain, a granulin domain, and an N-terminus signal peptide (Supplementary Fig. 3a). Phylogenetic analysis, which included sequences of Arabidopsis PLCP family proteins, indicated that this protein clustered with members of the RD21 subfamily (Supplementary Fig. 3b). Its sequence displayed the highest similarity (68.3%) to AtRD21A (Supplementary Fig. 3a). Consequently, we designated TraesCS2D02G513600 as TaRD21A. By analyzing the variation of TaRD21A in two populations, UP-RIL and BJ-DH, we identified two SNPs within TaRD21A. TaRD21A^C213T^ harbors a synonymous mutation, while TaRD21A^G286T^ carries a nonsynonymous mutation that leads to a alanine to serine (Ser-96) (Supplementary Fig. 3c, d). As a result, we designated TaRD21A in resistant cultivars (UC1110 and Jingshuang 16) as TaRD21A^R^ and in susceptible cultivars (PI610750 and Bainong 64) as TaRD21A^S^. Subsequently, TaRD21A^R^ fused to the C-terminus of RFP under the control of its native promoter (RD21Apro: TaRD21A^R^: RFP) to explore the subcellular localization. TaRD21A^R^: AtPIP1: RFP which previously shown to localize to the extracellular matrix^22^ was used as positive control. RD21Apro: TaRD21A^R^: RFP or RD21Apro: AtPIP1: RFP was transiently co-expressed with plasma membrane (PM) marker 35 S: AtPIP2A: RFP in *N. benthamiana* leaves by agroinfiltration. The results demonstrated that the fluorescence of TaRD21A^R^: RFP and RD21Apro: AtPIP1: RFP partially overlaps with that of AtPIP2A: RFP (Fig. 2a). When the cells were plasmolyzed by infiltration of 1.5 M sorbitol solution into the apoplast, RD21Apro: TaRD21A^R^: RFP and RD21Apro: AtPIP1: RFP separated from AtPIP2A-RFP-labeled PM and localized into apoplastic spaces (Fig. 2a). To further corroborate these findings, we extracted apoplastic fluid (AF) and total protein from *N. benthamiana* leaves expressing RD21Apro: TaRD21A^R^: RFP or RD21Apro: AtPIP1: RFP and conducted western blot assays to confirm their expression (Fig. 2b). The major bands from 70 to 95 KDa were observed on the blot indicating TaRD21A^R^: RFP was accumulated in the apoplastic spaces (Fig. 2b). In addition, the protease activity of PLCPs in AF extracted from *N. benthamiana* leaves expressing RD21Apro: TaRD21A^R^: RFP was investigated by ABPP assay using the protease activity-based probe DCG-04, which can be blocked by addition of E-64, a specific PLCP inhibitor^18^. Our results showed that the bands from 25–40 KDa could be observed on the blot in the ABPP assay. Furthermore, we expressed and purified recombinant TaRD21A^R^-GST from *E. coli* (Supplementary Fig. 3e). After a 2-h incubation with DCG-04, we also observed the major bands on blots from 25 to 40 KDa (Fig. 2d). As described in the previous studies^12^, the N- and C-terminus domain of PLCPs was removed, resulting in mature active PLCPs. In addition, RD21A with the granulin domain can also be active. Thus, the resulted bands on the blots which indicated protease activity profiles of PLCPs are highly polymorphic, with molecular masses ranging from 25 to 40 kDa. To investigate the function of TaRD21A^R^ protease activity in wheat resistance to WYMV infection, the conserved catalytic triad Cys-His-Asn among the PLCP family members^12^ was identified in TaRD21A^R^ (Supplementary Fig. 3a) and then substituted them with Glycine (TaRD21A^R^-M). Subsequently, we transiently expressed RD21Apro: TaRD21A^R^-M: RFP and RD21Apro: TaRD21A^R^: RFP in *N. benthamiana* leaves. Following a 48-h incubation, we inoculated the leaves with WYMV. At 7 dpi, we observed a significant reduction in cysteine proteinase activities of AF extracted from *N. benthamiana* leaves expressing RD21Apro: TaRD21A^R^-M: RFP compared to those expressing RD21Apro: TaRD21A^R^: RFP (Fig. 2e). As expected, the accumulation levels of WYMV CP in *N. benthamiana* leaves expressing RD21Apro: TaRD21A^R^: RFP were significantly lower than in those expressing RD21Apro: TaRD21A^R^-M: RFP (Fig. 2e), indicating that TaRD21A activity triggers the host plant resistance to WYMV.

### *TaRD21A*^*R*^ is involved in WYMV infection in wheat

**Fig. 3:**
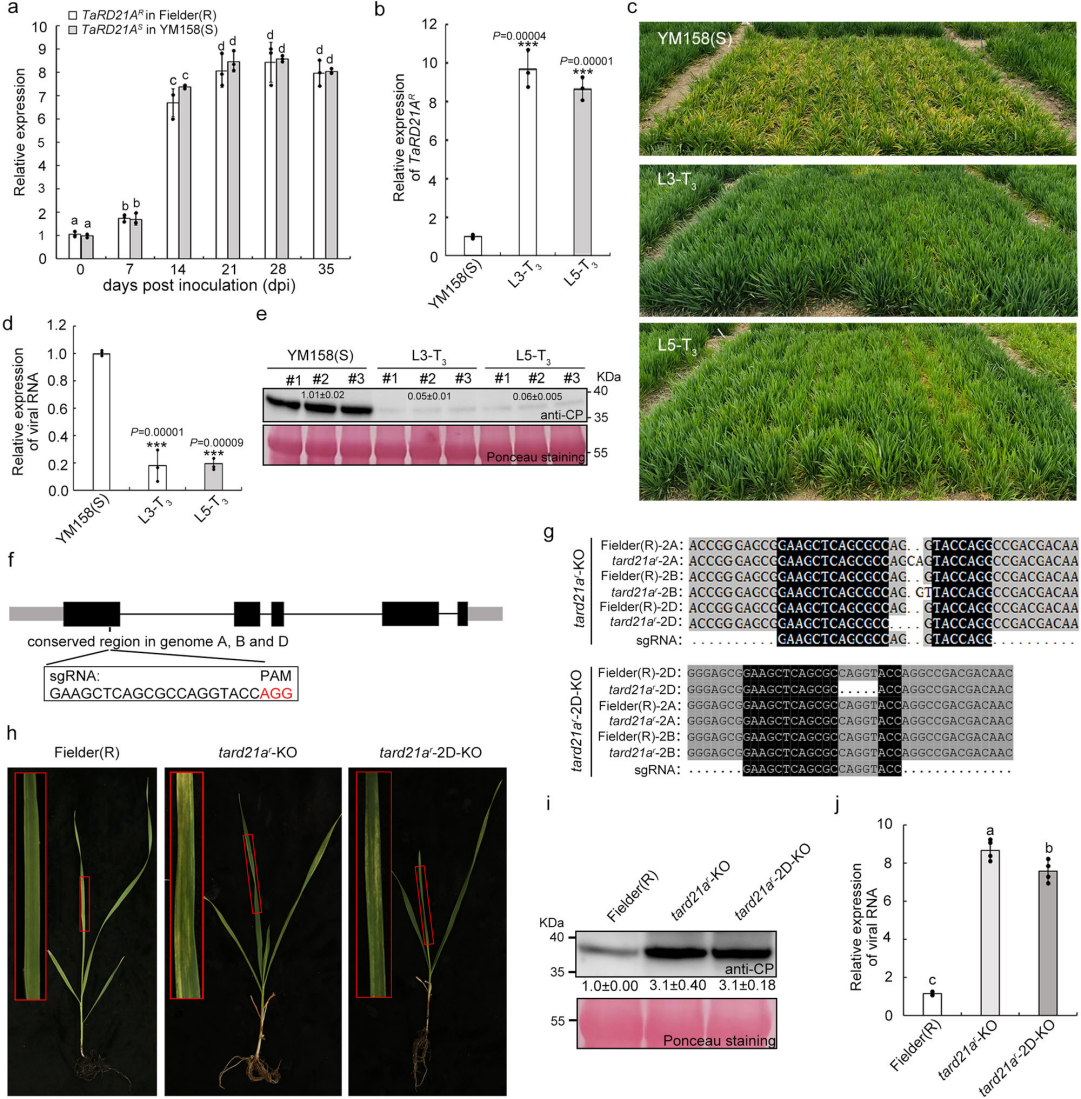
*TaRD21A*^*R*^ positively regulates wheat resistance to WYMV infection. **a** Relative expression levels of *TaRD21A*^*R*^ and *TaRD21A*^*S*^ in Fielder(R) and YM158(S) respectively at different times after WYMV inoculation which were determined by qRT-PCR. Values of qRT-PCR are the mean ± SD (one-way ANOVA with Tukey’s test, *n* = 3 biologically independent experiments, *P* values are shown in the Source Data file). **b** Transcript level of *TaRD21A*^*R*^ in two *TaRD21A*^*R*^ overexpression transgenic lines (L3-T_3_ and L5-T_3_) which determined by qRT-PCR and compared to that in YM158(S). Value is the mean ± SD (two-sided *t*-test, *n* = 3 biologically independent experiments, ***P < 0.001). **c** Assessment of L3-T_3_ and L5-T_3_ in a WYMV contaminated nursery at Yangzhou, Jiangsu Province, in 2022. YM158(S) was used as control. **d**, **e** The accumulation of viral RNA and protein in YM158(S), L3-T_3_ and L5-T_3_ plants from WYMV contaminated nursery, determined by qRT-PCR and western blot assay. Value is the mean ± SD (two-sided *t*-test, *n* = 3 biologically independent experiments, ***P < 0.001). Total RNA from YM158(S) in WYMV contaminated nursery was used as control. **f** Schematic showing the genomic location of *TaRD21A*. The sgRNA was designed to target all the three homoeoalleles (2 A, 2B and 2D) in wheat by CRISPR-Cas9 technology. **g** Three homoeoalleles sequence of *TaRD21A* in *tard21a*^*r*^-KO, *tard21a*^*r*^-2D-KO and Fielder(R). The sequence of *TaRD21A* on 2 A, 2B and 2D in *tard21a*^*r*^-KO contains a 2-bp insertion, 1-bp insertion and 4-bp deletion, respectively. The sequence of TaRD21A on 2D in tard21a^r^-2D-KO contains a 5-bp deletion. **h** The phenotype of Fielder(R), *tard21a*^*r*^-2D-KO and *tard21a*^*r*^-KO inoculated with WYMV at 40 dpi. **i**, **j** Detection of viral RNA and protein in the assayed wheat plants by qRT-PCR and western blot assays, respectively. Values of qRT-PCR are the mean ± SD (one-way ANOVA with Tukey’s test, *n* = 4 biologically independent experiments, *P* values are shown in the Source Data file). The data in (**e**) and (**i**) are representative of *n* = 3 independent experiments. Source data are provided as a Source Data file.

To investigate the expression level of *TaRD21A*, we utilized Fielder(R) and YM158(S) as host plants, both of which possessed the same genotype of TaRD21A as Bainong 64 and Jingshuang 16, respectively. Because of the high similarity in sequences between the copies of *TaRD21A* on chromosomes 2 A, 2B and 2D, their conserved region was selected for qRT-PCR assays (Supplementary Fig. 3f). As depicted in Fig. 3a, the transcript levels of TaRD21A in Fielder(R) and YM158(S) exhibited an approximately 8.0-fold induction from 0 to 21 dpi, which was sustained until 35 dpi. To further confirm the function of TaRD21A in wheat resistance to WYMV infection, we overexpressed *TaRD21A*^*R*^ in YM158(S) and generated *TaRD21A*^*R*^-OE transgenic wheat plants. Two positive T_0_ lines, *TaRD21A*^*R*^-L3-T_0_ and *TaRD21A*^*R*^-L5-T_0_, with high expression levels of *TaRD21A* (approximately 12-fold compared to control) were identified via qRT-PCR (Supplementary Fig. 4a). Two T_3_ lines, *TaRD21A*^*R*^-L3-T_3_ and *TaRD21A*^*R*^-L5-T_3_, displayed no obvious disease symptoms, with an induction of *TaRD21A*^*R*^ transcription as high as 10-fold compared to wild type (Fig. 3b, c). As anticipated, viral RNA and protein accumulation in these two lines were merely 0.2 and 0.1-fold, respectively, compared to those in YM158(S) (Fig. 3d, e). Additionally, we employed CRISPR/Cas9 technology to knockout *TaRD21A*^*R*^ in Fielder(R), employing an sgRNA targeting a conserved region common to the three copies of *TaRD21A* on chromosomes 2 A, 2B, and 2D (Fig. 3f). We successfully identified a *TaRD21A* knockout line where the D-genome copy alone was affected (*tard21a*^*r*^−2D-KO) and another knockout line where all three copies of mutant TaRD21A were disrupted (*tard21a*^*r*^-KO) via PCR/RE assay (Fig. 3g, Supplementary Fig. 4b). Sequencing of a gene highly resembling TaRD21A^R^ in *tard21a*^*r*^-KO and *tard21a*^*r*^−2D-KO confirmed that the editing construct did not complement this gene (Supplementary Fig. 4c). Following inoculation with WYMV, both *tard21a*^*r*^−2D-KO and t*ard21a*^*r*^-KO exhibited more severe mosaic patterns and stunted growth, accompanied by a significant increase in viral RNA and protein compared to Fielder(R) (Fig. 3h–j). Thus, our findings conclusively establish that TaRD21A acts as a positive regulator of wheat resistance to WYMV infection.

### TaRD21A^R^ induces immunity to WYMV in wheat through the release of a small peptide

**Fig. 4:**
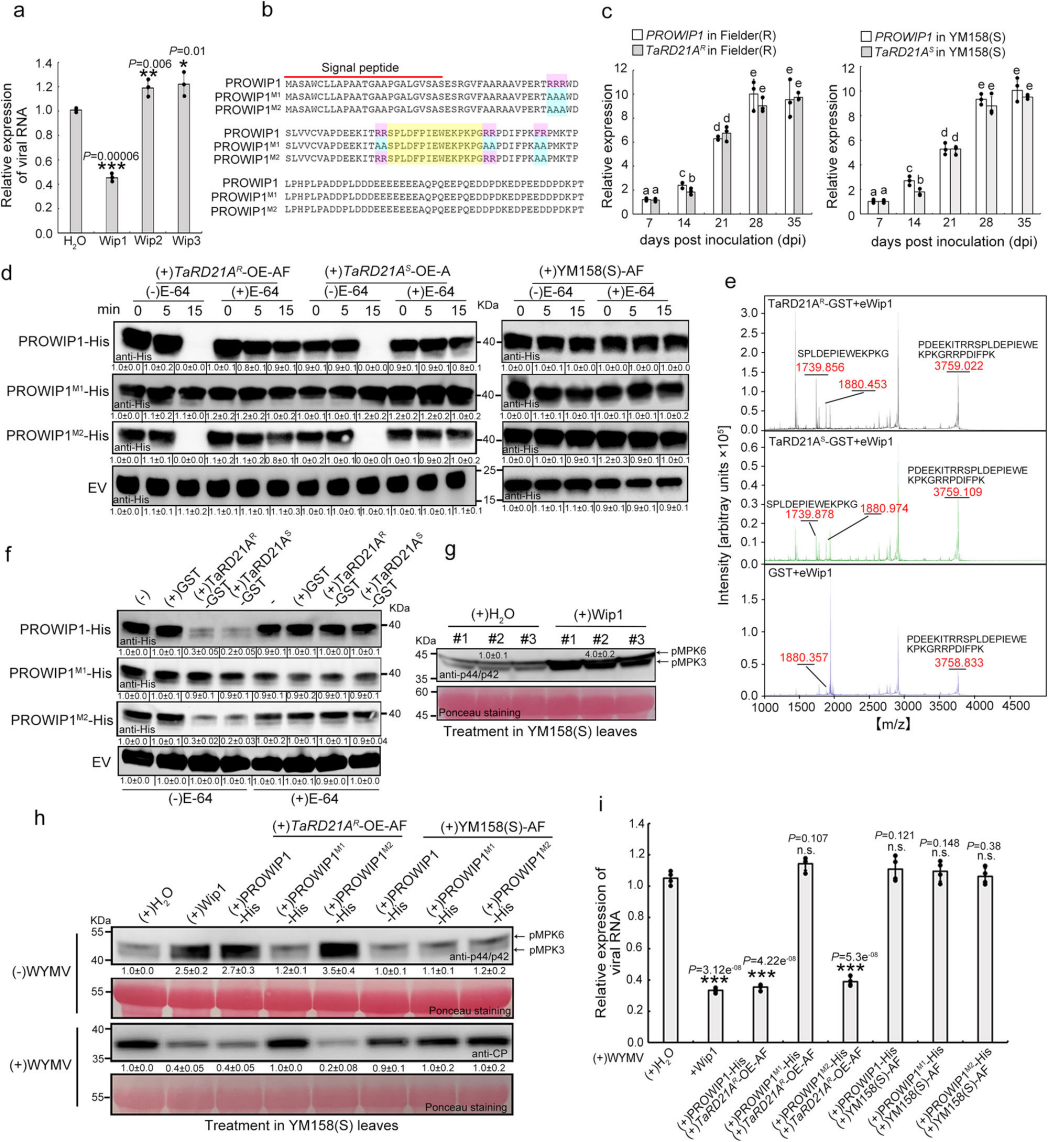
TaRD21A^R^-mediated release of Wip1 confers wheat resistance to WYMV infection. **a** The leaves of YM158(S) were infiltrated with candidate peptides and then inoculated with WYMV. The leaves treated with H_2_O were used as control. Values of qRT-PCR is the mean ± SD (two-sided *t-*test, *n* = 3 biologically independent experiments, ***P < 0.001, **P < 0.01, *P < 0.05). **b** Sequence alignment using PROWIP1 and its variant sequences. Putative cleavage sites (pink) were substituted with alanine (blue). Sequence of Wip1 in PROWIP1 and its variants are in yellow. The red line indicated the signal peptide. **c** The transcript level of *TaRD21A* and *PROWIP1* accumulation in the leaves of Fielder(R) or YM158(S) at different times after WYMV inoculation. Values of qRT-PCR is the mean ± SD (one-way ANOVA with Tukey’s test, *n* = 3 biologically independent experiments, *P* values are shown in the Source Data file). **d** In vitro cleavage assays using PROWIP1, PROWIP1^M1^ and PROWIP1^M2^-His as substrates with or without E-64, respectively. PROWIP1-His, PROWIP1^M1^-His, and PROWIP1^M2^-His were individually incubated with AF from the leaves of *TaRD21A*^*S*^-OE, *TaRD21A*^*R*^-OE transgenic line or YM158(S). Western blot assays were done using an anti-His antibody. **e** MALDI-TOF analysis of eWip1 cleavage by TaRD21A^R^-GST and TaRD21^S^-GST. Masses of eWip1 and mature Wip1 are indicated. The GST purified protein was used as control. **f** Cleavage of PROWIP1-His, PROWIP1^M1^-His, and PROWIP1^M2^-His by TaRD21A^R^-GST or TaRD21^S^-GST. Cleavage was detected by western blot assay using His antibody. EV in (**d**) and (**f**) is representative of expressing vector which containing His tag. **g** Wip1 activated MAPK signaling in vivo. Leaves infiltrated with H_2_O were used as controls. **h** In vitro released Wip1 is active in vivo. The wheat leaves infiltrated with H_2_O was used as control. **i** The accumulation of viral RNA in the infiltrated leaves of YM158(S) was confirmed by qRT-PCR assay at 7 dpi. Asterisks indicate significant differences between each treatment. Values of qRT-PCR is the mean ± SD (two-sided *t*-test, *n* = 4 biologically independent experiments, ***P < 0.001, n.s., no significant.). The data in (**d**, **f**–**h**) are representative of *n* = 3 independent experiments. Source data are provided as a Source Data file.

PLCPs was involved in small peptides maturation, thereby activating systemic immunity^12,23^. In line with this knowledge, we extracted AF from the leaves of *TaRD21A*^*R*^-OE lines. Subsequently, we identified three small peptide candidates (Wip1, Wip2, and Wip3) in AF and synthesized them individually (Supplementary Data 7). These peptides were then infiltrated into wheat leaves, followed by WYMV inoculation (Supplementary Fig. 5a). At 5 dpi, we observed a remarkable reduction of approximately 60% in the accumulation of viral RNA specifically in Wip1-treated wheat leaves when compared to control plants (Fig. 4a). Further investigation led us to discover a sequence [(N)-SPLDFPIEWEKPKPG-(C)] within the peptide, which, upon searching in the wheat genome database (https://plants.ensembl.org/), revealed a gene encoding a precursor protein designated as PROWIP1 (Accession No. TraesCS6B02G251800.1). PROWIP1 comprises 140 amino acids, commencing with a signal peptide at its N-terminus and featuring five Arg-Arg or Phe-Arg sequence motifs (RR/FR motifs) that represent potential cleavage sites for PLCPs^17^ (Fig. 4b). The expression pattern of *PROWIP1* closely mirrored that of *TaRD21A* in Fielder(R) and YM158(S) under WYMV infection (Fig. 4c). To discern the role of TaRD21A activity, we engineered the overexpression of *TaRD21A*^*S*^ in YM158(S), resulting in the creation of *TaRD21A*^*S*^-OE lines. Subsequently, we expressed and purified recombinant TaRD21A^R^-GST and TaRD21A^S^-GST (Supplementary Fig. 3e). Then, the protease activity of these recombinant proteins and AF extracted from the leaves of *TaRD21A*^*R*^-OE and *TaRD21A*^*S*^-OE lines were monitored by ABPP assay using DCG-04 (Supplementary Fig. 5b, c). To investigate whether TaRD21A cleaved Wip1 from PROWIP1, TaRD21A^R^-GST, TaRD21A^S^-GST, and AF from all assayed leaves were examined using fluorescent substrates containing RR/FR motifs, unequivocally confirming that RR/FR motifs were indeed the efficient cleavage sites (Supplementary Fig. 5d). Moreover, we engineered two modified versions of PROWIP1 with RR/FR substitutions. In the modified protein PROWIP1^M1^, all six RR motifs were substituted with AA motifs, while in PROWIP1^M2^, the predicted cleavage sites, except for two RR motifs flanking Wip1, were substituted with AA motifs (Fig. 4b). Subsequently, we expressed and purified recombinant PROWIP1-His, PROWIP1^M1^-His, and PROWIP1^M2^-His from *E. coli* (Supplementary Fig. 5e). These proteins were co-incubated with AF extracted from the leaves of *TaRD21A*^*R*^-OE, *TaRD21A*^*S*^-OE lines, and YM158(S), respectively. Western blot assays using His antibody confirmed that PROWIP1-His and PROWIP1^M2^-His were indeed cleaved by components within AF from the leaves of the *TaRD21A*^*R*^-OE and *TaRD21A*^*S*^-OE lines, but not by those from YM158(S). PROWIP1^M1^-His remained unaffected by components within the AF from all materials (Fig. 4d). Furthermore, cleavage activity towards PROWIP1-His and PROWIP1^M2^-His was observed using AF from the leaves of Fielder(R) with WYMV infection, while no such activity was detected in AF from *tard21a*^*r*^-KO leaves or Fielder without WYMV infection (Supplementary Fig. 6a). To directly investigate the role of TaRD21A in the maturation of the Wip1 peptide, the activity of recombinant TaRD21A^R^-GST and TaRD21A^S^-GST was analyzed by MALDI-TOF assay using a synthetic, extended Wip1 peptide (eWip1) as substrate. This extended peptide included eight precursor-derived amino acids surrounding the mature Wip1 sequence. The results demonstrated that eWip1 was processed in a TaRD21A^R^ or TaRD21A^S^-dependent manner, resulting in the production of the mature peptide (Fig. 4e, Supplementary Fig. 6b). Furthermore, in line with the cleavage activity observed towards PROWIP1 and its altered proteins using AF extracted from wheat leaves, PROWIP1-His and PROWIP1^M2^-His, but not PROWIP1^M1^-His, were cleaved by TaRD21A^R^-GST and TaRD21A^S^-GST in vitro (Fig. 4f, Supplementary Fig. 6c). In addition, to gain deeper insights into Wip1 function in wheat immunity, we conducted transcriptome analyses in the leaves of YM158(S) treated with Wip1. This analysis identified a total of 1169 differentially expressed genes, predominantly associated with MAPK signaling (Supplementary Fig. 7). This finding was further substantiated through western blotting using an anti-phospho p44/p42 antibody (Fig. 4g). Additionally, we performed a large-scale cleavage assay, followed by the extraction of small peptides (<10 kDa)^18^. This assay revealed an increase in the phosphorylation of MAP kinases in the leaves of YM158(S) in response to PROWIP1 or PROWIP1^M2^ peptide fractions when incubated with AF from the leaves of *TaRD21A*^*R*^-OE lines, compared to incubation with AF from YM158(S). Conversely, the inclusion of PROWIP1^M1^ peptide fractions failed to induce the phosphorylation of MAP kinases (Fig. 4h). Subsequently, we infiltrated the leaves of YM158(S) with these peptide fractions, followed by WYMV inoculation. At 7 dpi, we observed a significant reduction, approximately 70% and 80%, in the accumulation of viral RNA and protein, respectively, in leaves treated with PROWIP1 or PROWIP1^M2^ peptide fractions incubated with AF from the leaves of *TaRD21A*^*R*^-OE, as compared to leaves treated with AF from YM158(S) (Fig. 4h, i). Moreover, when leaves were infiltrated with PROWIP1 or PROWIP1^M2^ protein fractions incubated with AF from the leaves of Fielder(R) with WYMV infection, we observed an induction of MAP kinase phosphorylation and a reduction in viral RNA and protein accumulation. These effects were absent when using AF from *tard21a*^*r*^-KO lines with WYMV infection (Supplementary Fig. 6d, e). To further explore the function of PROWIP1 cleavage by TaRD21A^R^, we co-expressed PROWIPpro: PROWIP1: His, PROWIPpro: PROWIP1^M1^: His, or PROWIPpro: PROWIP1^M2^: His with RD21Apro: TaRD21A^R^: RFP, followed by inoculation with WYMV in *N. benthamiana*. Similar to the effects observed with Wip1 treatment, an increase in MAP kinase phosphorylation and a reduction in viral protein accumulation were only observed in leaves co-expressing PROWIPpro: PROWIP1: His or PROWIPpro: PROWIP1^M2^: His and RD21Apro: TaRD21A^R^: RFP. The inclusion of a MAPK signal inhibitor prevented the increase in MAPK kinase phosphorylation and restored viral protein accumulation in these plants (Supplementary Fig. 8). In summary, our results strongly suggest that the cleavage of PROWIP1 by TaRD21A plays a critical role in MAPK signaling and contributes significantly to wheat resistance against WYMV infection.

### WYMV NIa interferes with TaRD21A activity

**Fig. 5:**
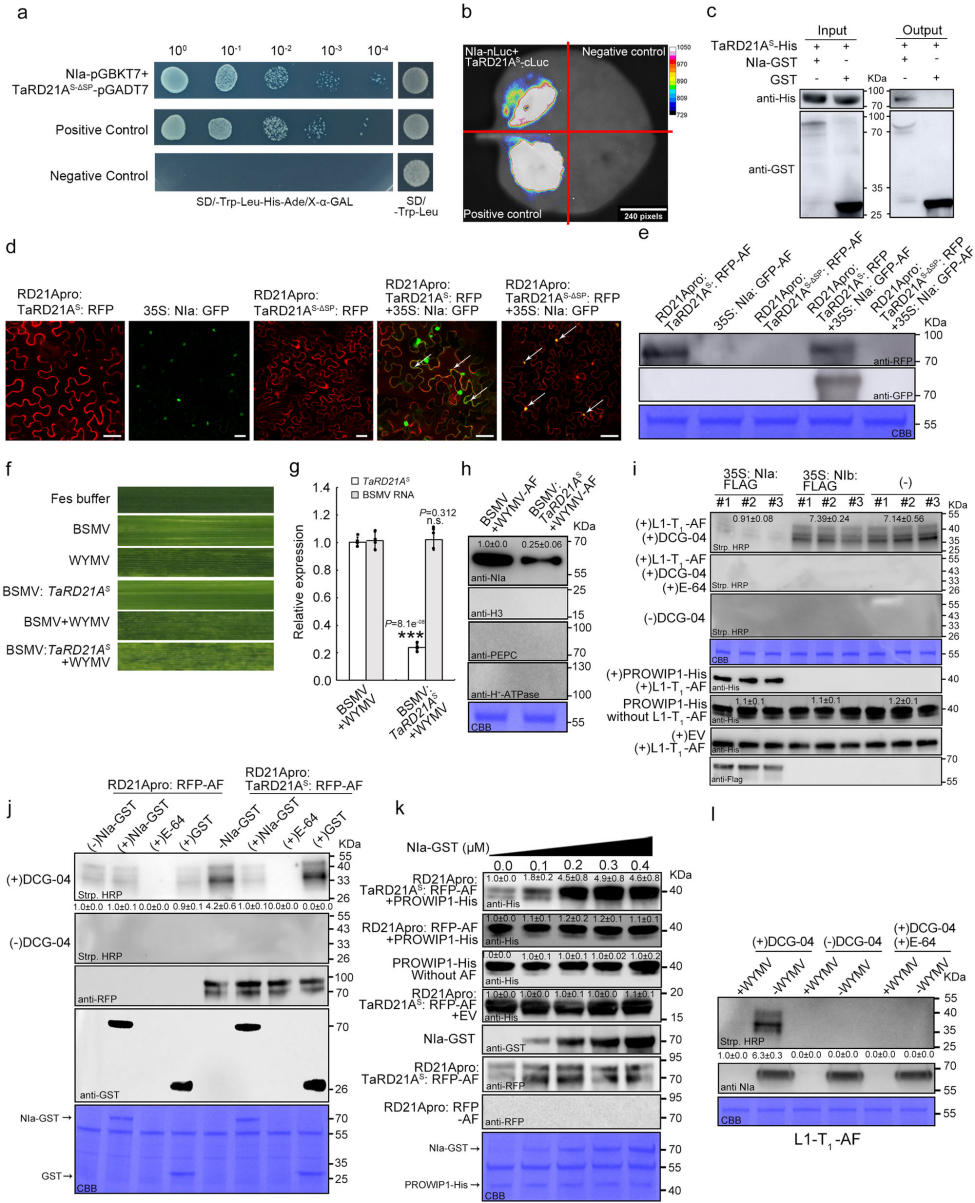
NIa interacts with TaRD21A^S^ to affect its protease activity. **a** The interaction between NIa and TaRD21A^S^ was confirmed by Y2H assays. **b** LCI assay was used to confirm the interaction between NIa-nLuc and TaRD21A^S^-cluc. Leaf cells co-expressing sGF-nLuc and cLuc-SAR or nLuc and TaRD21A^S^-cluc were used as positive or negative controls, respectively. **c** GST pull-down assay was used to detect the interaction between NIa-His and TaRD21A^S^-GST. **d** Subcellular distribution of NIa: GFP, TaRD21A^S-ΔSP^: RFP and TaRD21A^S^: RFP in *N. benthamiana* epidermal cells. Three times each experiment was repeated independently with similar results. Bar, 50 μm. **e** Accumulation of 35 S: NIa: GFP, RD21Apro: TaRD21A^S-ΔSP^: RFP and RD21Apro: TaRD21A^S^: RFP in AF from assayed plants in (**d**) was determined by western blot assays using GFP or RFP antibody. **f** Mild chlorotic mosaic symptoms were observed at 14 dpi in the fourth leaves of the plants inoculated with Fes buffer (Mock), WYMV, BSMV, BSMV: *TaRD21A*^*S*^, BSMV + WYMV and BSMV: *TaRD21A*^*S*^ + WYMV, respectively. **g** Accumulation of *TaRD21A* and BSMV RNA in the leaves of YM158(S) co-infected with BSMV + WYMV or BSMV: *TaRD21A*^*S*^ + WYMV. Values of qRT-PCR is the mean ± SD (two-sided *t*-test, *n* = 4 biologically independent experiments, ***P < 0.001, n.s., no significant). **h** AF was extracted from the leaves of YM158(S) infected with BSMV + WYMV and BSMV: *TaRD21A*^*S*^ + WYMV for western blot assay using NIa specific antibody. H3, PEPC and H^+^-ATPase antibody was used to verify the absence of nuclear, cytoplasmic, or plasma membrane components. **i** The effect of NIa on TaRD21A^S^ activity. The AF from wheat leaves with or without NIb expression were used as control. **j**, **k** Effect of NIa-GST on the cysteine proteases activity in AF from *N. benthamiana* leave expressing RD21Apro: TaRD21A^S^: RFP or RD21Apro: RFP. **l** The effect of WYMV infection on TaRD21A^S^ activity. (−)DCG-04 indicated AF without DCG-04 labelling and used for distinguish from background signals. EV in (**i**–**k**) is representative of expressing vector which containing His tag. Coomassie Blue staining in (**e**, **h**–**l**) shows the assayed protein loaded. The data in (**c**, **e**, **h**–**l**) are representative of *n* = 3 independent experiments. Source data are provided as a Source Data file.

To unravel how WYMV bypasses TaRD21A-mediated wheat immunity in susceptible cultivars, we conducted an extensive investigation. Firstly, we assessed the interaction between WYMV proteins and TaRD21A^S-ΔSP^, an altered TaRD21A^S^ protein lacking its signal peptide. Among the 11 WYMV proteins examined, only NIa exhibited a discernible interaction with TaRD21A^S-ΔSP^, as confirmed by Y2H assays (Fig. 5a, Supplementary Fig. 9). This interaction was further validated through in vivo and in vitro experiments, including split luciferase complementation (SLC) and pull-down assays (Fig. 5b, c). Subsequently, we transiently expressed 35 S: NIa: GFP, RD21Apro: TaRD21A^S^: RFP, and RD21Apro: TaRD21A^S-ΔSP^: RFP in *N. benthamiana* leaves to facilitate AF extraction. Western blot assays clearly demonstrated that the accumulation of 35 S: NIa: GFP occurred in the apoplastic space when co-expressed with RD21Apro: TaRD21A^S^: RFP (Fig. 5d, e). Furthermore, we employed the BSMV-mediated gene silencing system to knock down *TaRD21A*^*S*^ in YM158(S). Wheat seedlings were inoculated with WYMV, BSMV, BSMV: TaRD21A^S^, BSMV + WYMV, or BSMV: TaRD21A^S^ + WYMV. After 14 dpi, qRT-PCR results revealed a significant reduction in the accumulation of *TaRD21A*^*S*^, but not BSMV RNA, in wheat seedlings inoculated with BSMV: TaRD21A^S^ + WYMV (Fig. 5f, g). Subsequently, at 40 dpi, the amount of NIa in AF isolated from BSMV: TaRD21A^S^ + WYMV-treated plants were reduced by 80% compared to that in BSMV + WYMV-treated plants (Fig. 5h). We also transiently expressed 35 S: NIa: FLAG and 35 S: NIb: FLAG in the leaves of *TaRD21A*^*S*^-L1-T_1_ or YM158(S) using particle bombardment-mediated transformation for AF extraction. Leaves expressing or not expressing NIb were used as controls. The PLCP protease activity and the cleavage of PROWIP1 in AF extracted from TaRD21A^S^-L1-T_1_ leaves was blocked by expressing 35 S: NIa: FLAG, unlike the control plants (Fig. 5i, Supplementary Fig. 10a). Additionally, PLCP activity in AF from *N. benthamiana* leaves transiently expressing RD21Apro: TaRD21A^S^: RFP was inhibited when co-incubated with recombinant NIa-GST (Fig. 5j). The influence of NIa on the cleavage of PROWIP1-His by AF from *N. benthamiana* leaves transiently expressing RD21Apro: TaRD21A^S^: RFP was found to be dependent on the dosage of NIa-GST (Fig. 5k, Supplementary Fig. 10b). Similarly, the reduction in TaRD21A^S^ protease activity was confirmed by incubating TaRD21A^S^-GST with NIa-MBP in vitro (Supplementary Fig. 10c). In addition, the leaves of *TaRD21A*^*S*^-L1-T_1_ were inoculated with WYMV, PLCP activity in AF extracted from these leaves was significantly reduced at 40 dpi (Fig. 5l). In conclusion, our findings suggest that NIa acts as an inhibitor of TaRD21A.

### Phosphorylation of TaRD21A^R^ interferes with its interaction with NIa

**Fig. 6:**
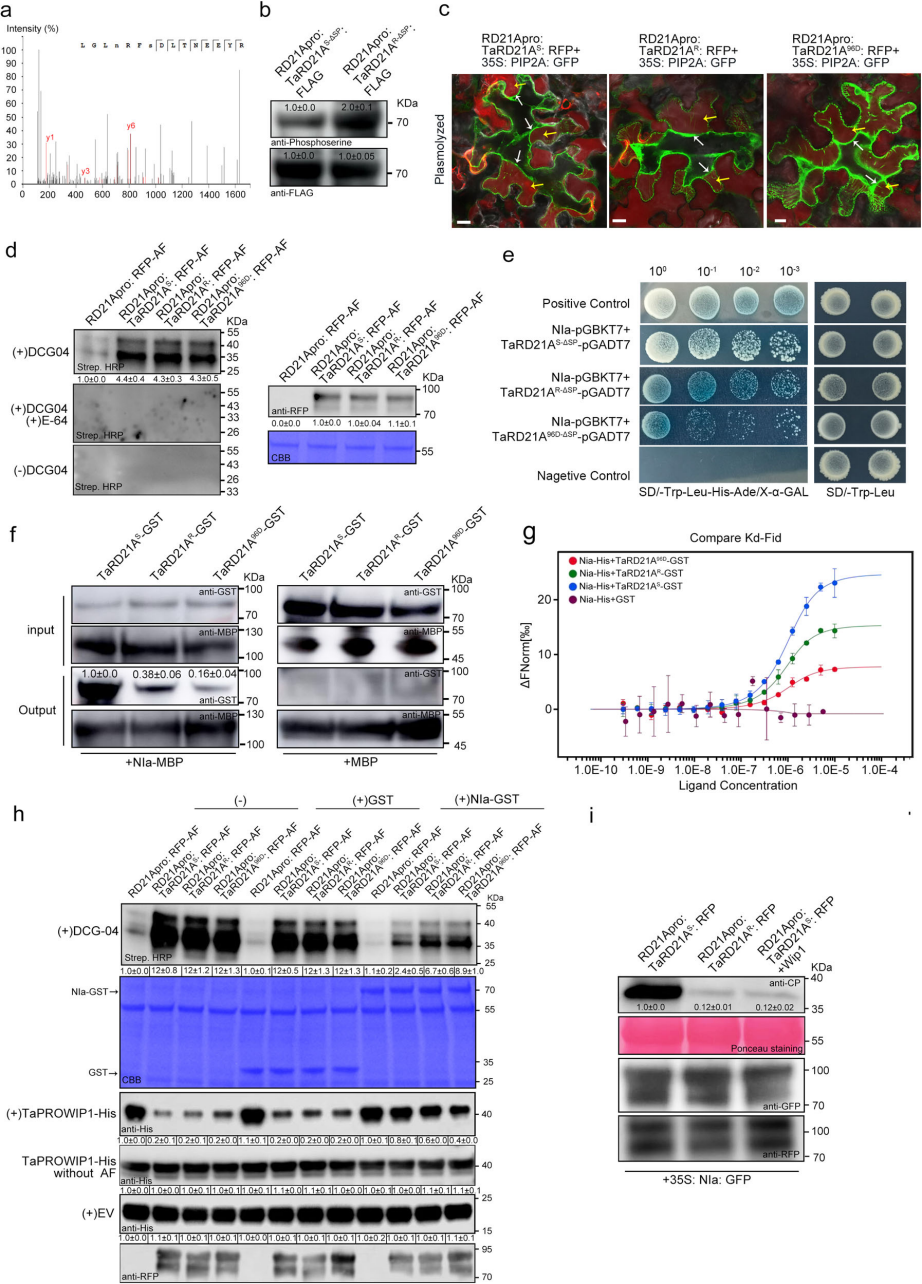
Phosphorylation of TaRD21A^R^ at Ser-96 is crucial for the interaction between NIa and TaRD21A. **a** Phosphorylation of Ser-96 in TaRD21A^R^ detected by LC-MS/MS. **b** The phosphorylation levels of TaRD21A^R-ΔSP^: FLAG and TaRD21A^S-ΔSP^: FLAG was determined through western blot analysis using a phosphoserine specific antibody. **c** Subcellular localization of RD21Apro: TaRD21A^S^: RFP, RD21Apro: TaRD21A^R^: RFP, and RD21Apro: TaRD21A^96D^ in *N. benthamiana* leaf cells. Bar, 10 μm. Three times each experiment was repeated independently with similar results. **d** The presence of TaRD21A^S^: RFP, TaRD21A^R^: RFP and TaRD21A^96D^: RFP in AF were determined by western blot assay using an RFP antibody. AF were labeled with DCG-04 in the presence or absence of E-64, followed by western blot assay for determining the activity of AF from the assayed *N. benthamiana* leaves in (**c**). RD21Apro: RFP was used as control. (−)DCG-04 indicated that the AF without a non-probe control and used for distinguish from background signals. **e** Interactions between NIa and TaRD21A^S-ΔSP^, TaRD21A^R-ΔSP^ or TaRD21A^96D-ΔSP^ in a Y2H assay. **f** Analysis of the Nia-MBP interaction with TaRD21A^S^-GST, TaRD21A^R^-GST or TaRD21A^96D^-GST by pull-down assay. MBP empty vector was used as a negative control. **g** Microscale thermophoresis assay was used to detect NIa-His binding affinity to TaRD21A^S^-GST, TaRD21A^R^-GST or TaRD21A^96D^-GST. Nia-His together with GST was used as negative control. Each binding assay was repeated three times independently, and bars represent SD. Data points are represented as means ± SD. **h** The effect of NIa on protease activity in AF were extracted from *N. benthamiana* leaves transiently expressing RD21Apro: TaRD21A^S^: RFP, RD21Apro: TaRD21A^R^: RFP or RD21Apro: TaRD21A^96D^: RFP. RD21Apro: RFP was used as control. **i** The *N. benthamiana* leaves co-expressed RD21Apro: TaRD21A^S^: RFP or RD21Apro: TaRD21A^R^: RFP and 35 S: NIa: GFP, followed by WYMV inoculation in present or absent Wip1 treatment. Coomassie Blue staining in (**d**, **h**) shows the assayed protein loaded. The data in (**b**, **d**, **f**, **h**, **i**) are representative of *n* = 3 independent experiments. Source data are provided as a Source Data file.

To investigate the impact of phosphorylation on TaRD21A, we extracted proteins from the protoplasts of YM158(S) expressing RD21Apro: TaRD21A^R-ΔSP^: FLAG to identify phosphorylated residues through LC-MS. Our analysis confirmed the phosphorylation of TaRD21A^R-ΔSP^ at Ser-96 (Fig. 6a). Furthermore, Western blot assays revealed that the phosphorylation level of TaRD21A^R-ΔSP^: FLAG was increased by 2.0-fold when compared to TaRD21A^S-ΔSP^: FLAG expressed in the protoplasts of WY158(S) (Fig. 6b). To mimic phosphorylation, we substituted Ser-96 with aspartic acid, creating TaRD21A^96D^. We then transiently co-expressed RD21Apro: TaRD21A^S^: RFP, RD21Apro: TaRD21A^R^: RFP or RD21Apro: TaRD21A^96D^: RFP with 35 S: AtPIP2A: RFP in *N. benthamiana* leaves followed by infiltration of 1.5 M sorbitol solution into the apoplast. Under confocal microscopy, all forms of TaRD21A localized into apoplastic spaces (Fig. 6c, Supplementary Fig. 11a). Western blot and ABPP assay further confirmed that the accumulation levels of these TaRD21A variants and proteinase activities were similar across all AF from *N. benthamiana* leaves transiently expressing RD21Apro: TaRD21A^S^: RFP, RD21Apro: TaRD21A^R^: RFP and RD21Apro: TaRD21A^96D^: RFP (Fig. 6d). We also expressed and purified recombinant TaRD21A^96D^-GST (Supplementary Fig. 11b). The proteinase activity also showed no significant changes between TaRD21A^S^-GST, TaRD21A^R^-GST, and TaRD21A^96D^-GST (Supplementary Fig. 11c). We proceeded to investigate the effect of Ser-96 phosphorylation on the interaction between TaRD21A and NIa. Y2H assays demonstrated that TaRD21A^S-ΔSP^, as well as TaRD21A^R-ΔSP^ and TaRD21A^96D-ΔSP^, interact with NIa (Fig. 6e). This finding was also supported by results from MST and pull-down assays (Fig. 6f, g). Next, we examined the effect of NIa on TaRD21A activity. The results of ABPP assay indicated that NIa-GST had a little effect on the proteinase activity in AF from *N. benthamiana* leaves transiently expressing RD21Apro: TaRD21A^96D^: RFP, slightly influenced those expressing RD21Apro: TaRD21A^R^: RFP, but significantly reduced that expressing RD21Apro: TaRD21A^S^: RFP (Fig. 6h). Moreover, in the presence of NIa, the cleavage of AF from *N. benthamiana* leaves transiently expressing RD21Apro: TaRD21A^S^: RFP to PROWIP1 was strongly inhibited, while the cleavage of those expressing RD21Apro: TaRD21A^R^: RFP, and RD21Apro: TaRD21A^96D^: RFP was only slightly affected (Fig. 6h, Supplementary Fig. 11d). These results were further confirmed using TaRD21A^S^-GST, TaRD21A^R^-GST, and TaRD21A^96D^-GST in vitro (Supplementary Fig. 11e). To explore the role of NIa in TaRD21A-mediated wheat resistance, we co-expressed RD21Apro: TaRD21A^S^: RFP or RD21Apro: TaRD21A^R^: RFP with 35 S: NIa: GFP in *N. benthamiana* leaves followed by WYMV infection. At 7 dpi, viral protein accumulation in *N. benthamiana* leaves co-expressing RD21Apro: TaRD21A^R^: RFP and 35 S: NIa: GFP was only 0.1-fold compared to that in leaves co-expressing RD21Apro: TaRD21A^S^: RFP and 35 S: NIa: GFP. However, Wip1 treatment in *N. benthamiana* leaves co-expressing RD21Apro: TaRD21A^S^: RFP and 35 S: NIa: GFP also significantly reduced WYMV protein accumulation (Fig. 6i). In conclusion, these findings suggest that the phosphorylation of TaRD21A impacts its interaction with NIa and subsequently influences the proteinase activities of TaRD21A.

### Natural variations in TaRD21A are responsible for subspecies-specific resistance to WYMV infection

**Fig. 7:**
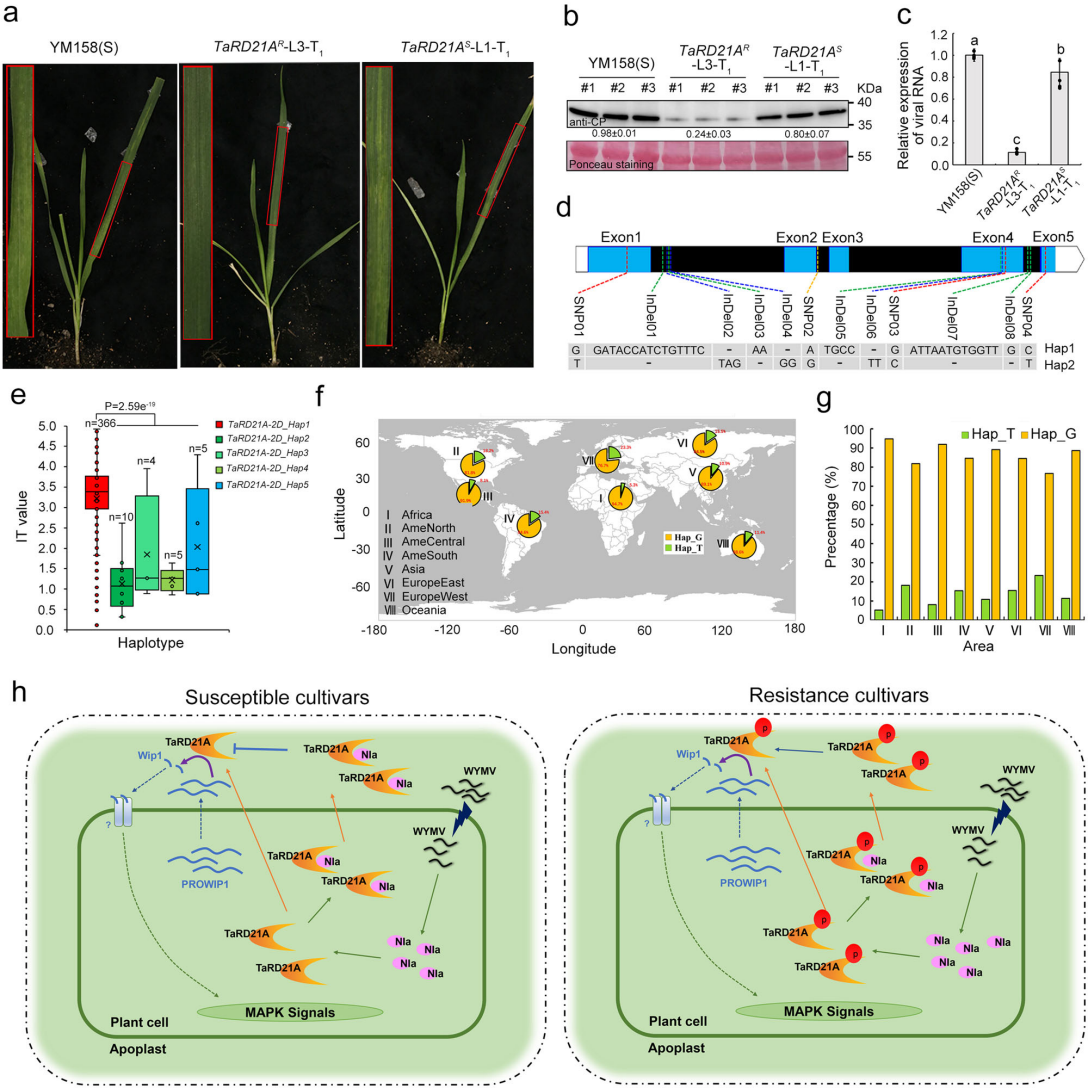
Natural variants of *TaRD21A* confer wheat resistance to WYMV infection. **a** The phenotype of *TaRD21A*^*R*^-L3-T_1_ and *TaRD21A*^*S*^-L1-T_1_ inoculated with WYMV at 40 dpi. **b**, **c** Detection of viral RNA and protein in the assayed wheat plants by qRT-PCR and western blot assays using CP specific primer and antibody, respectively. Values of qRT-PCR are the mean ± SD (one-way ANOVA with Tukey’s test, *n* = 4 biologically independent experiments, *P* values are shown in the Source Data file). **d** Polymorphic distribution of *TaRD21A* coding regions. **e** Haplotype analysis of *TaRD21A-2D* in the test wheat accessions using the identified 12 SNPs/InDels. For box-plot, the horizontal lines from top to bottom represent the maximum, first quartile, median, third quartile, and minimum of the total data, respectively. The cross in the middle of the box represents the average. n, represent the number of wheat accessions with the corresponding haplotype. Statistics: for both datasets, two-sided *t* test was performed. *n* the number of wheat accessions with the corresponding haplotype. **f**, **g** Geographic distribution of accessions carrying susceptible (Hap_G) and resistant (Hap_T) alleles of *TaRD21A-2D*. **h** A working model illustrating TaRD21A-mediated wheat disease resistance. In the susceptible cultivars, TaRD21A is exported into the leaf apoplast to release the signal peptide from its precursor which then activates MAPK signals. However, the WYMV encoded protein NIa interferes with the activity of TaRD21A when they interact with each other and move to the leaf apoplast. In the resistant cultivars, phosphorylation of TaRD21A reduces the interaction with NIa and avoids the inhibition of TaRD21A activity by NIa in the leaf apoplast, resulting in higher resistance to WYMV infection. The data in (**b**) are representative of *n* = 3 independent experiments. Source data are provided as a Source Data file.

To confirm the role of the amino acid change at Ser-96 in wheat resistance to WYMV infection, we inoculated the positive T_1_ lines of *TaRD21A*^*R*^-OE and *TaRD21A*^*S*^-OE (*TaRD21A*^*R*^-L3-T_1_ and *TaRD21A*^*S*^-L1-T_1_) with WYMV, using YM158(S) as a control. At 40 dpi, *TaRD21A*^*S*^-L1-T_1_, as well as YM158(S), exhibited more severe mosaic patterns and stunted growth, along with a significant induction of viral RNA and protein, compared to *TaRD21A*^*R*^-L3-T_1_ (Fig. 7a–c). Furthermore, the positive T_2_ line (*TaRD21A*^*R*^-L4-T_2_) with low expression levels of *TaRD21A* was inoculated with WYMV. At 40 dpi, no disease symptoms were detected, similar to the results observed for *TaRD21A*^*R*^-L3-T_2_ (Supplementary Fig. 12a). Western blot and qRT-PCR assays revealed that *TaRD21A*^*R*^-L4-T_2_ exhibited a reduction of approximately 70% in viral RNA and 80% in CP compared to YM158(S) (Supplementary Fig. 12b, c). In addition, we transiently expressed RD21Apro: TaRD21A^R^ in the resistant cultivar UC1110 through particle bombardment-mediated transformation and then inoculated the leaves with WYMV (Supplementary Fig. 12d). At 7 dpi, the expression level of *TaRD21A*^*R*^ was induced 8.0-fold in the leaves transiently expressing RD21Apro: TaRD21A^R^. However, the amount of viral RNA remained unchanged in the leaves expressing RD21Apro: TaRD21A^R^ compared to the control plants (Supplementary Fig. 12e). We conducted *t* tests based on the phenotypes of the progeny with resistant alleles and susceptible alleles in the UP-RIL population and BJ-DH population. The results showed that progeny with resistant alleles exhibited significantly higher resistance to WYMV than those with susceptible alleles (Supplementary Fig. 13). Additionally, phenotypic analysis of varieties with resistant alleles and susceptible alleles from the two association panels indicated that the varieties with resistant alleles displayed greater resistance to WYMV (Supplementary Fig. 13). Furthermore, we sequenced the three homoeologous copies of *TaRD21A* in 406 wheat accessions and identified significantly different haplotypes in terms of WYMV resistance (Fig. 7d, e, Table 1, Supplementary Fig. 14a, b). *TaRD21A-2D*, in particular, showed four nonsynonymous SNPs (including G286T) and eight InDels forming five main haplotypes (Fig. 7d, Table 1). Wheat accessions carrying *TaRD21A-2D_Hap1* exhibited significantly weaker resistance to WYMV compared to accessions carrying the other four haplotypes (Fig. 7e). Further analysis indicated that the combination of superior haplotypes from the three homoeologous copies of *TaRD21A* could significantly enhance WYMV resistance (Supplementary Fig. 14c). An evaluation of the global distribution of the *TaRD21A* resistance allele in 1168 wheat germplasms^24–27^ from all over the world revealed that the frequencies of wheat accessions with the *TaRD21A* resistant allele (Hap_T) ranged from only 5.3% in Africa to 23.3% in Europe West (Fig. 7f, g).

**Table 1:** Haplotype analysis of *TaRD21A-2D* in the test wheat accessions using the identified 12 SNPs/InDels

Haplotype	SNP01	Indel01	Indel02	Indel03	Indel04	SNP02	Indel05	Indel06	SNP03	Indel07	Indel08	SNP04	IT value	Number of varieties
Hap1	G	+	-	+	-	A	+	-	G	+	+	C	3.22	366
Hap2	T	-	+	-	+	G	-	+	C	-	-	T	1.13	10
Hap3	T	-	+	-	+	A	+	-	G	-	-	T	1.85	4
Hap4	T	-	+	-	+	A	+	-	C	-	-	T	1.22	5
Hap5	T	-	+	-	+	G	+	-	C	-	-	T	2.03	5
	Ala96Ser	Indel	Indel	Indel	Indel	Spelice	Frameshift	Frameshift	Glu387Asp	Indel	Indel	Arg442Cys		

## Discussion

Soil-borne viruses transmitted through the root parasite *P. graminis* have inflicted significant yield losses in global winter wheat cultivation for many years. While the development and utilization of resistant cultivars represent eco-friendly and effective strategies to combat these diseases, the identification of loci associated with virus resistance in wheat has remained scarce until now^9^. In this investigation, we have mapped the genes responsible for WYMV resistance in wheat, pinpointing TaRD21A, a member of the PLCP gene family, as a key player in the defense against this virus. PLCP genes hold critical roles in plant defense mechanisms, particularly in combating fungal pathogens^12^. However, their role in plant antiviral response has largely remained uncharted territory. Our study outcomes provide compelling evidence that PLCPs are indeed instrumental in plant antiviral responses. To demonstrate this, we achieved stable overexpression of *TaRD21A* in wheat, resulting in heightened resistance to WYMV infection. Conversely, when we employed CRISPR/Cas9-based technology to knockout *TaRD21A* in wheat, susceptibility to infection increased significantly (Fig. 3). These findings strongly indicate that PLCPs play a pivotal role in plant antiviral defenses. Two potential mechanisms have been proposed for the role of PLCPs in mediating host defense responses: (i) the proteolytic shedding of extracellular receptor domains^28^ and (ii) the release of small signal peptides from their precursors^18^. Our study reveals that both PROWIP1-His and PROWIP1^M2^-His can be cleaved not only by components within AF from the leaves of the *TaRD21A*^*R*^-OE and *TaRD21A*^*S*^-OE lines but also by TaRD21A^S^-GST and TaRD21A^R^-GST recombinant proteins (Fig. 4d–f). These findings were further substantiated by employing AF from the leaves of Fielder(R) and *tard21a*^*r*^-KO with WYMV infection (Supplementary Fig. 7a). Given that the expression level of *TaRD21A* significantly increased in Fielder(R) following WYMV infection, PROWIP1-His and its altered protein remained unaltered when exposed to AF from the leaves of Fielder(R) and YM158(S) without WYMV infection (Fig. 4d, Supplementary Fig. 7a). These results suggest that TaRD21A can mediate the release of the Wip1 peptide. Moreover, Wip1 treatment significantly reduced the WYMV RNA accumulation (Fig. 4a) and the cleavage of PROWIP1 by AF from the leaves of the *TaRD21A*^*R*^-OE and Fielder(R) plays a central role in wheat resistance to WYMV infection (Fig. 4h, i; Supplementary Fig. 7d, e). Thus, we conclude that the role of TaRD21A in wheat resistance to WYMV relies on its catalytic activity in generating a small peptide that facilitates plant immunity.

The apoplast, encompassing intercellular spaces and plant cell walls, serves as a critical site for interactions between plants and pathogens, where specialized infection structures of many fungal or oomycete pathogens are established^29,30^. Despite plant viruses being primarily intracellular parasites, certain proteins involved in plant antiviral responses, such as S-adenosyl homocysteine hydrolase (SAHH) and plasma membrane protein PtBP1, have been identified in the apoplast of virus-infected leaves^31,32^. In our current investigation, we have uncovered that TaRD21A is secreted into the apoplast, where it plays a pivotal role in wheat antiviral response. These findings underscore the significance of the plant apoplast as a vital arena for mounting plant antiviral defenses. Numerous small peptides in the apoplast have been identified as key signaling molecules initiating plant immune responses^33^. During plant-pathogen interactions, small peptides exhibit antimicrobial properties, directly interacting with distinct bacterial cell wall structures or intracellular organelles^34^. Additionally, they engage with multiple plasma membrane-localized receptors to activate or modulate plant immunity^35^. Our study yielded evidence of reduced WYMV infection following Wip1 treatment (Fig. 4a), implicating small peptides in plant antiviral responses. Recent research has demonstrated that small peptides induce the generation of reactive oxygen species (ROS) and upregulate the expression of plant defense-related genes, thus enhancing host resistance to viral infections^36^. In our investigation, we demonstrated that the TaRD21A-mediated release of the Wip1 peptide activates MAPK signaling, a conclusion supported by Wip1 treatment (Fig. 4g, h, Supplementary Fig. 7a, b). Prior studies have underscored the pivotal role of the MAPK cascade in plant antiviral defense^37,38^. For instance, MAPKs directly phosphorylate viral nucleoproteins, triggering immunity against cross-kingdom infections such as barley yellow striate mosaic virus in host plants and its insect vectors^39^. Consequently, we postulate that the small signal peptide released through TaRD21A activity activates plant immunity against viral infections through this pathway, suggesting that wheat varieties carrying TaRD21A^R^ may also exhibit resistance to other viral diseases.

While plant viruses are primarily intracellular parasites, several viral components have been detected in the extracellular space^40,41^. However, the mechanisms by which viral proteins infiltrate intercellular regions remain largely unknown. Our findings confirmed that WYMV NIa was transported into the apoplastic space through its interaction with TaRD21A (Fig. 5d, e). The quantity of NIa was significantly reduced in the AF isolated from *TaRD21A*-silenced plants (Fig. 5f–h). These results suggest that viral proteins may leverage host factors for transmembrane transport. Furthermore, it is worth noting that PLCP activities in various subfamilies can be counteracted by specific pathogen effectors, including viral proteins^20,21^. In tomato, for instance, natural Rcr3 variants diminished interactions with the fungal effector protein Avr2, preventing the inhibition of Rcr3 activity by Avr2^42,43^. Our results align with these reports and demonstrate that the natural variation in TaRD21A can disrupt the interaction between NIa and TaRD21A, ultimately enhancing TaRD21A activity in resistant wheat varieties (Fig. 6d–h). Thus, we propose that natural variations in proteases play pivotal roles in the ongoing arms race between plants and pathogens.

In crop breeding endeavors, disease resistance and grain yield represent two paramount objectives. Several reports have suggested that high levels of crop resistance may come at the cost of reduced yield^44,45^. However, many natural disease resistance alleles in crop plants provide robust disease resistance without observable setbacks in plant growth and yield. For example, the rice ROD1 resistance allele offers broad-spectrum disease resistance across multiple pathogens without affecting agronomic traits^46^. Additionally, a natural allele of the rice C2H2-type transcription factor controls rice nonrace-specific resistance to blast disease without discernible impacts on growth and yield^47^. Overall, we contend that natural resistance alleles stand as promising candidates in crop breeding initiatives aimed at bolstering both yields and disease resistance.

In this study, we identified a natural variant of TaRD21A in WYMV-resistant wheat cultivars. This variant can be phosphorylated in vivo (Fig. 6a, b). Phosphorylation plays a critical role in gene expression, signal transduction, protein subcellular localization, and protein-protein interactions^48^. However, our investigation revealed that phosphorylation at Ser-96 had no discernible effect on TaRD21A protease activity or subcellular localization (Fig. 6c, d). Furthermore, our field test results demonstrated that wheat varieties carrying TaRD21A^R^ exhibited markedly higher resistance to WYMV infection compared to those carrying TaRD21A^S^ (Fig. 7d, e; Table 1). This indicates that this natural TaRD21A variant specifically governs wheat resistance to WYMV without affecting other functions of TaRD21A. Additionally, we found that this *TaRD21A* allele is present in more than 1000 wheat varieties from various countries (Fig. 7f, g). Consequently, we believe that this natural TaRD21A variant holds substantial potential for integration into wheat breeding projects focused on enhancing resistance to WYMV.

In summary, we present a working model illustrating the role of TaRD21A in wheat resistance to WYMV (Fig. 7h). The expression of *TaRD21A* is significantly upregulated upon WYMV infection, and the resulting TaRD21A protein is transported to the apoplastic space. Subsequently, TaRD21A protease activity liberates Wip1 from PROWIP1, initiating MAPK signaling and activating wheat resistance against WYMV infection. In one scenario, TaRD21A from susceptible wheat cultivars is recognized by the WYMV-encoded NIa protein. This interaction leads to the translocation of NIa-TaRD21A complexes into the apoplastic spaces, where NIa inhibits TaRD21A activity, thus suppressing host immunity mediated by TaRD21A. Conversely, in another scenario, resistant cultivars possess a natural TaRD21A variant that is phosphorylated at Ser-96. This phosphorylation prevents its interaction with NIa, avoiding inhibition of its activity by NIa in the leaf apoplast, leading to strong resistance to WYMV infection. These insights enhance our understanding of the molecular mechanisms underlying wheat resistance to WYMV infection and offer valuable implications for wheat breeding initiatives focused on enhancing resistance to WYMV.

## Methods

### Plant materials

Two association panels were used for GWAS analysis. Panel I consisted of 243 wheat accessions from the Yellow and Huai River valleys as described by Zhang et al.^49^. Panel II consisted of 163 historical and current Chinese wheat cultivars as described by Chen et al.^50^. All plants were grown in a WYMV nursery field in Xiping (33.40° N, 113.3° E) in individual plots during 2016 to 2017 with two replications in the growing seasons 2017–2018 and 2018–2019. An F_10_ recombinant inbred line (RIL) population resulting from a cross of UC1110 and PI610750 (UP-RIL) composed of 187 lines and a doubled haploid (DH) population resulting from a cross of Bainong 64 and Jingshuang 16 (BJ-DH) composed of 181 lines were generated for linkage mapping. The two populations were planted in the same field in Xiping in the 2017–2018 and 2018–2019 growing seasons, with two replications. As a control, the highly susceptible variety Xinmai 18 was planted every 10th row. Disease severity was investigated at the jointing stage when infection of Xinmai 18 had reached the level 3 infection type (IT)^51^. All the tested materials grew well. The best linear unbiased prediction (BLUP) value and broad-sense heritability were calculated using the lme4 package of R software on the basis of a mixed linear model.

### Genotyping and GWAS

The 243 wheat accessions of Panel I were genotyped using the Wheat 660 K SNP array^49,51,52^ and the 163 accessions of Panel II were genotyped using the Wheat 90 K SNP array^50,53^. After quality control, only SNPs with minor allele frequency (MAF) > 0.05 and missing data <20% in the association panel were kept for GWAS analysis using PLINK software as described in Sun et al. and Purcell et al.^53,54^. The GWAS analysis was implemented using the GAPIT package of R software and was based on a mixed linear model (PCA + K)^55^. The threshold of the significant *P* value was set as 1.0E^−4^ for Panel I and 1.0E^−3^ for Panel II for all replications. Haploview 4.2 was used to analyze haplotypes for significant SNPs. Collinearity analysis of all the genes in the specific intervals among the subgenomes of hexaploid wheat was performed using jcvi. compa. synteny module of MCScan (Python) software^56^.

### Bulk segregant analysis (BSA)

The WYMV-resistant and WYMV-susceptible pools (three biological replications each) were generated by combining 10 resistant lines (each resistant pool) and 10 susceptible lines (each susceptible pool) and were further genotyped using the Wheat 55 K (BJ-DH population) and 660 K (UP-RIL population) SNP arrays for BSA, respectively. The number and chromosomal positions of different SNPs between each pair of resistant and susceptible pools were analyzed using RStudio software to preliminarily map loci that modulate WYMV resistance^57^.

### Linkage mapping and QTL detection

The UP-RIL population was genotyped using 1494 markers consisting of three types of markers (251 simple sequence repeats [SSRs], 15 expressed sequence tags [ESTs] and 1228 diversity array technologies [DArTs]). Based on previous genome resequencing results of UC1110 and PI 610750, 27 additional markers were developed, including 1 cleaved amplified polymorphic sequence (CAPS), 8 kompetitive allele-specific PCR (KASP) and 15 insertion/deletion (InDel) markers. The BJ-DH population was genotyped using the wheat 55 K SNP array, and a total of 13613 SNPs were used for QTL mapping. Linkage mapping was performed using IciMapping 4.1, and nnTwoOpt and SAD were used to complete the linkage algorithm and to perform standard measurements^58^. The degree of phenotypic variance explained (PVE) was used to evaluate the genetic effects of the identified QTLs. The threshold of the logarithm of odds (LOD) score was set to 2.5 to indicate the presence of a QTL.

### Bulked segregant RNA sequencing of the biparental RIL population

Three resistant pools (IT < 1) and three susceptible pools (IT > 4) of the UP-RIL population were used for bulked segregant RNA sequencing (BSR-seq). Each WYMV-resistant or WYMV-susceptible pool was composed of an equivalent number of leaves from 10 lines of the UP-RIL population. The sampled leaves were flash frozen in liquid nitrogen and then stored at −80 °C for BSR-seq. In addition, we integrated the published gene expression profile database of wheat (http://202.194.139.32/expression/wheat.html), and the gene was considered to be expressed when its expression abundance log_2_(TPM + 1) ≥ 0.5^59^.

### WYMV inoculation in laboratory and field

Wheat cv. Yangmai 158 (Susceptible cultivar, S) was used to investigate the roles of *TaRD21A* and small signal peptides in WYMV infection. YM158(S) and wheat cv. Fielder (Resistant cultivar, R) was used to generate *TaRD21A* overexpression (*TaRD21A*^*R*^-OE, *TaRD21A*^*S*^-OE) and *TaRD21A* knockout transgenic lines (*tard21a*^*r*^-KO, *tard21a*^*r*^-2D-KO). The WYMV infectious clones pCB-SP6-R1 and pCB-SP6-R2 were obtained from a published source and were used to infect wheat plants^60^. The inoculated plants were grown in a growth chamber maintained at 8 °C. For field assessments, the *TaRD21A*^*R*^-OE lines were grown in a nursery in Yangzhou, China from 2019 to 2022.

### RNA extraction, qRT-PCR and western blot assays

Total RNA was extracted from tissue samples using TRIzol^TM^ Reagent (Invitrogen, Carlsbad, CA, USA). First-strand cDNA was synthesized using a reverse-transcription PCR (RT-PCR kit) (Promega, Madison, WI, USA), random primers, and 1 μg of total RNA per 20 μL reaction mixture. Quantitative PCR was performed with SYBR Green qRT-PCR mixture (Vazyme, Nanjing, China) on an ABI7900HT Sequence Detection System (Applied Biosystems, Foster City, CA, USA)^61^. The qRT-PCR expression data were normalized to those of the wheat *Actin* gene and analyzed using the 2^−ΔΔCT^ method^62^. All the primers used in this study are listed in Supplementary Data 8. For western blot assay, total protein was extracted from tissue tissues using a protein extraction kit (Solarbio, Beijing, China). The resulting samples were analyzed by SDS-PAGE. The resulting blotted nitrocellulose membranes were incubated in blocking buffer (0.05% Tween-20 and 5% nonfat milk powder in Tris buffer with Tween-20) followed by probing with the specific primary and secondary antibodies (1:10000, anti-Mouse and Rabbit, Abbkine Scientific Co., California, USA, A21010 and A21020).

### Analysis of the *TaRD21A* sequence

The full-length *TaRD21A* sequence was retrieved from the wheat genome database (http://plants.ensembl.org/index.html) and then amplified by RT-PCR from wheat cv. Jingshuang 16 (resistant cultivars) and Bainong 64 (susceptible cultivars) via specific primers (Supplementary Data 8), followed by sequencing. The confirmed *TaRD21A* sequence was further analyzed using ORF Finder software (https://www.ncbi.nlm.nih.gov/orffinder/). The conserved domains in the *TaRD21A* sequence were identified using Pfam (http://pfam.xfam.org/), InterProScan (http://www.ebi.ac.uk/interpro/search/sequence-search), and ScanProsite (http://prosite.expasy.org/scanprosite/) software. Sequences of Arabidopsis PLCP family proteins were retrieved from The Arabidopsis Information Resource database (https://www.arabidopsis.org/). The sequences of the proteins were aligned using DNAMAN 7.0 software (Lynnon Biosoft, USA) and were used to construct a phylogenetic tree using MEGA 7.0 software^63^.

### Transient expression in plants

To investigate the subcellular localization patterns of TaRD21A^R^, TaRD21A^S^, TaRD21A^96D^ and WYMV NIa, full-length *TaRD21A* or its variants and *NIa* sequences were amplified by RT-PCR via specific primers (Supplementary Data 8). AtPIP1, which was previously shown to localize in the extracellular matrix, was used as a positive control. The resulting sequences were inserted into pGWB454 (RFP-tag) and pGWB505 (GFP-tag) using the gateway method to generate 35 S: TaRD21A: RFP, 35 S: TaRD21A^S^: RFP, 35 S: TaRD21A^R^: RFP or 35 S: TaRD21A^96D^: RFP, 35 S: AtPIP1: RFP and 35 S: NIa: GFP expression vectors, respectively. Then, the upstream 2012 bp promoter region of TaRD21A^R^ (same to that of TaRD21A^S^) was cloned and inserted into 35 S: TaRD21A: RFP and its variants, 35 S: AtPIP1: RFP and an RFP-containing empty vector with an RD21A promoter instead of the 35 S promoter to generate RD21Apro: TaRD21A^S^: RFP, RD21Apro: TaRD21A^R^: RFP, RD21Apro:TaRD21A^96D^: RFP, RD21Apro: RFP and RD21Apro: AtPIP1. *Agrobacterium tumefaciens* GV3101 cells were transfected with the constructs, allowed to grow, and then infiltrated into *N. benthamiana* leaves. The 1.5 M sorbitol solution was used for plasmolysis and AtPIP2A (AT3G53420) was used as the plasma membrane (PM) marker proteins^64^. To investigate the colocalization of NIa and TaRD21A^S^ or TaRD21A^S-ΔSP^ (an altered TaRD21A^S^ protein whose signal peptide was removed), *Agrobacterium* cultures harboring RD21Apro: TaRD21A^S-ΔSP^: RFP or RD21Apro: TaRD21A^S^: RFP and 35 S: NIa: RFP were grown, mixed to a 1:1 ratio, and then infiltrated into *N. benthamiana* leaves. The AF was extracted from these assay leaves for western blot assay using GFP (1: 5000, TransGen Biotech, Beijing, China, HT801-01) and RFP antibody (1:5000, Abbkine Scientific Co, California, USA, Cat. No. ABM40169). To confirm the function of TaRD21A^R^ protease activity in wheat resistance to WYMV infection, the conserved catalytic triad Cys-His-Asn in TaRD21A was identified^12^ and then substituted it with Glycine (TaRD21A^R^-M). RD21Apro: TaRD21A^R^-M: RFP was generated and then transiently expressed in *N. benthamiana* leaves, respectively, followed by inoculation with WYMV. To investigate the function of TaRD21A in the cleavage of PROWIP1, the coding sequence of ProWIP1 was cloned into pGWB405 (His-tag) using the gateway method to produce 35 S: ProWIP1: RFP. Instead of the 35 S promoter, the promoter of PROWIP1 was cloned into ProWIP1: RFP to produce ProWIPpro: PROWIP1: His. *Agrobacterium* cultures harboring RD21Apro: TaRD21A^R^: RFP or ProWIPpro: PROWIP1: His were coinfiltrated into *N. benthamiana* leaves, which were then inoculated with WYMV. *N. benthamiana* coexpressing RD21Apro: TaRD21A^R^: RFP and ProWIPpro: PROWIP1: His were treated with 10 μM U0126 (Absin, Shanghai, China) solution to inhibit MAPK signaling during WYMV infection. At seven days after infection, all the leaves were sampled for Western blot assays using CP (1:2000, prepared by Huaan Biotechnology Co., Hangzhou, Zhejiang, China, and stored in our lab), RFP, His (1:5000, TransGen Biotech, Beijing, China, Cat. No. HT501-01) or phospho-p44/p42-specific antibody (1:5000, Cell Signaling Technology, Massachusetts, USA, Cat. No. #4370). For particle bombardment assays, recombinant plasmids containing the TaRD21A^R^ or NIa coding sequence with its natural promoter (RD21Apro:TaRD21A^R^ and 5 S: NIa: FLAG) were prepared. Fourteen-day-old leaves of the resistant cultivar UC1110 or *TaRD21A*^*S*^ transgenic overexpression lines (*TaRD21A*^*S*^-L1-T_1_) were directly bombarded using He/1100 particles (Bio-Rad, Hercules, CA, USA) at a bombardment distance of 6 cm. After bombardment, the wheat plants were incubated at room temperature for 6-hour under darkness. The bombarded leaves were subsequently inoculated with WYMV and then incubated at 8 °C under a 16 h/8 h (light/darkness) photoperiod. At 7 days post inoculation (dpi), the inoculated leaves were sampled for RNA or protein extraction. To investigate the phosphorylation of TaRD21A^R^ at Ser-96, TaRD21A^R^ without signal peptide (TaRD21A^R-ΔSP^) was fused to FLAG and expressed under its native promoter in the protoplast from the leaves of YM158(S). Then, the fusion protein was purified using FLAG beads (Sigma-Aldrich, Shanghai, China Cat. No. A2220) for LC-MS assay and western blot assay using FLAG (1: 5000, TransGen Biotech, Beijing, China, Cat. No. HT201-01) and phosphoserine-antibody (1: 5000, Sigma-Aldrich, Shanghai, China, Cat. No. SAB5200086).

### Generation of transgenic wheat lines

To produce transgenic wheat plants overexpressing *TaRD21A*, the full-length *TaRD21A* sequence from YM158(S) and Fielder(R) was cloned behind the ubiquitin promoter in a pUbi:00 vector to produce pUbi: TaRD21A^S^ and pUbi: TaRD21A^R^. A pAHC20 vector carrying a selective bar gene was cotransformed together with pUbi: TaRD21A^S^ or pUbi: TaRD21A^R^ into immature embryos of YM158(S) by particle bombardment^65^. The resulting T_0_ to T_3_ transgenic lines were selected via PCR using ubiquitin promoter-specific primers (Supplementary Data 8). To knock out *TaRD21A*, sgRNAs specific for the three *TaRD21A* copies were designed. A sgRNA-clustered, regularly interspaced, short palindromic repeats (CRISPR)-CRISPR-associated 9 (Cas9) expression vector was used for transforming immature embryos of the wheat cultivar Fielder. Positive transgenic seedlings were identified using a PCR/restriction enzyme (PCR/RE) assay^66^. Briefly, genomic DNA was extracted from the transgenic plants and amplified via PCR by use of a high-fidelity DNA polymerase and primers specific for *TaRD21A*-*2A*, *TaRD21A*-*2B*, and *TaRD21A*-*2D*. The resulting amplicons including the gRNA target site were digested by *Kpn*I restriction enzymes. The presence of undigested products indicated the loss of the *Kpn*I site from within the original *TaRD21A* sequences. The loss of the *Kpn*I site was further confirmed by sequencing. The primers used are listed in Supplementary Data 8.

### Apoplastic fluid (AF) isolation

To prepare AF, leaves from wheat or *N. benthamiana* plants subjected to various treatments were submerged in water within a vacuum chamber for 30 min at 400 mbar. Subsequently, these drained leaves were carefully positioned inside the barrel of a 50 mL syringe, aligning the leaf edges with the barrel ends. The barrel, with the needle hub pointing downward, was then placed into a 50 mL centrifuge tube. This assembly was subjected to centrifugation for 20 min at 2000×g and 4 °C. Following centrifugation, the resulting AF was extracted from the tube and promptly stored at −20 °C. All assayed AF from the leaves of wheat or *N. benthamiana* plants was verified the absence of nuclear, cytoplasmic, or plasma membrane components by western blot assay using H3 (1: 5000, Cell Signaling Technology, Massachusetts, USA, Cat. No. #4499), PEPC and H^+^-ATPase antibody (1: 5000, Amyjet Scientific, Wuhan, China, Cat. No. AS09-458 and AS07-260) (Supplementary Fig. 15).

### Identification and functional analysis of TaRD21A-released small peptides

To identify small peptides released by TaRD21A, AF was isolated from *TaRD21A*^*R*^-OE transgenic wheat lines and enriched for small peptides in these fluids using 10 kDa Amicon centrifugation filters (EMD Millipore). Briefly, 5 mL of apoplastic fluid, containing approximately 4.5 mg of total protein, was taken from each sample and centrifuged to enrich peptides <10 kDa. Formic acid (FA) and acetonitrile (ACN) were added to these samples such that the final concentration was 0.5% each, followed by mass spectrometry^18^. The tandem mass spectrometry (MS/MS) data were analyzed using MASCOT software 2.3.02 against the contents of the database with known contaminants (incorporated in MASCOT), and the identified peptide sequences were subsequently used to search the *T. aestivum* sequences within the database (http://plants.ensembl.org/Triticum_aestivum/Info/Index). To investigate the functions of the identified peptides, the second leaf of YM158(S) at two-week-old was infiltrated with a mock solution or a 5 μM peptide solution via needle-less syringes. Six hours later, the infiltrated leaves were harvested and then analyzed for MAPK phosphorylation through western blotting using a phospho-p44/p42-specific antibody or were inoculated with WYMV.

### Protein interaction experiments

For pull-down assays, the coding sequences of NIa, TaRD21A or its derivatives were subcloned into pGEX4T-2, pMAL-c2X or pET-32a expression vectors containing GST-, MBP- or His-tag sequences by using specific primers (Supplementary Data 8). The resulting GST, His or MBP fusion constructs were then expressed in *Escherichia coli* (BL21). GST- or MBP-tagged proteins together with 100 mL of glutathione agarose or MBP beads (GE Healthcare, Beijing, China) were incubated for 2-h at 4 °C. The beads were washed five times with PBS buffer and then incubated with an equal amount of bacterial lysates containing His-tagged or GST-tagged protein for another 2-h at 4 °C. After washes for five times, MBP-tagged, His-tagged or GST-tagged proteins were detected by western blot assays in conjunction with His, MBP or GST antibodies (1:5000, TransGen Biotech, Beijing, China, Cat. No. HT701-01, HT601-01). All yeast two-hybrid (Y2H) assays were carried out according to the Clontech Yeast Protocols Handbook^67^. Interaction between SV40 large T-antigen (T) and murine p53 (53) was used as the positive control, while interaction between SV40 large T-antigen (T) and human lamin C (Lam) was used as the negative control. For firefly luciferase complementation imaging (LCI) assays, the coding sequences of NIa and TaRD21A were amplified and inserted into pCambia1300-nLuc or pCambia1300-nLuc to produce NIa-nluc and TaRD21A-cluc, respectively. *Agrobacterium* cells carrying NIa-nluc and an equal amount of *Agrobacterium* cells carrying TaRD21A-cluc were then mixed together and infiltrated into leaves of *N. benthamiana*. After three days, the same leaves were infiltrated again with a 0.2 mM luciferin (Thermo Scientific, USA) solution followed by the detection of luciferase activity via a low-light cooled charge-coupled device (CCD) imaging apparatus (NightOWL II LB983 with indiGO software). The interaction between NIa and TaRD21A or its derivatives was also confirmed by microscale thermophoresis assay (MST) performed with a Monolith NT.115 kit following the manufacturer instructions (NanoTemper Technologies, Munich, Germany). Specifically, the purified NIa protein was labeled via a Monolith NT protein labeling kit and then incubated together with TaRD21A or its derivatives. The interaction of the mixed protein samples was analyzed using NanoTemper analytical software to determine the equilibrium dissociation constant (KD).

### BSMV-mediated gene silencing

A specific fragment of *TaRD21A*^*S*^ containing *Not*I and *Pac*I restriction sites was obtained by RT-PCR, and BLAST analysis of the wheat genome databases (https://urgi.versailles.inra.fr/blast/) revealed that this fragment was not similar to any other gene. The capped in vitro transcripts of BSMV RNAα, RNAβ, and RNAγ were prepared from linearized plasmid DNAs (pBSMV-α, pBSMV-β and pBSMV-γ) via a Message T7 in vitro transcription kit (Ambion, Austin, TX; Promega, Shenzhen, China) following the manufacturers’ instructions. Second leaves of YM158(S) at two-leaf-stage were infected with BSMV (BSMV:γ, BSMV:TaRD21A^S^). The third leaf of each plant was inoculated with WYMV at 7 dpi. At 21 dpi, AF was isolated from virus-infected leaves for western blot assays via NIa-specific antibodies.

### Activity-based protein profiling (ABPP) assay

To investigate TaRD21A activity, ABPP was performed using the DCG-04 as fluorescent probe. In details, AF were extracted from the *TaRD21A*^*R*^-OE, *TaRD21A*^*S*^-OE or the leaves of *N. benthamiana* plants transiently expressing *TaRD21A* and subsequently labeled in a solution consisting of 2 μM DCG-04, 50 mM sodium acetate (pH 5.5), and 1 mM dithiothreitol (DTT) at room temperature. For in vitro assay, the concentration of purified TaRD21A^R^-GST and TaRD21A^S^-GST was adjusted to 0.2 mg ml^−1^ with 15 mM sodium phosphate buffer, pH 6.0, 0.2 mM DTT and then preincubated with 5 μM E-64 (Sigma-Aldrich, Shanghai, China) in a total volume of 200 μL for 30 min at room temperature prior to the addition of 0.2 μL of 2 mM DCG-04. For inhibition assays, AF were first incubated together with 0.0, 0.1, 0.2, 0.3 or 0.4 μM purified NIa protein and then treated with 2 μM DCG-04 for 40 min in presence or absence of E-64. The reactions were stopped by the addition of 10 μL of 5× SDS-PAGE sample buffer. The resulting samples were analyzed via SDS-PAGE. Proteins attached to the nitrocellulose membranes were detected using streptavidin-conjugated horseradish peroxidase (HRP; 1:3000, Abbkine Scientific Co, California, USA, Cat. No. A21000). As fluorescent intensity of bound probe reflects activity the signal was quantified using ImageJ Software.

### Protease activity profiling

To investigate TaRD21A activity, AF were extracted from the *TaRD21A*^*R*^-OE, *TaRD21A*^*S*^-OE or the leaves of *N. benthamiana* plants transiently expressing *TaRD21A* and subsequently labeled in a solution consisting of 2 μM DCG-04, 50 mM sodium acetate (pH 5.5), and 1 mM dithiothreitol (DTT) at room temperature. For in vitro activity assay, the concentration of purified TaRD21A^R^-GST and TaRD21A^S^-GST was adjusted to 0.2 mg ml^−1^ with 15 mM sodium phosphate buffer, pH 6.0, 0.2 mM DTT and then preincubated with 5 μM E-64 (Sigma-Aldrich, Shanghai, China) in a total volume of 200 μL for 30 min at room temperature prior to the addition of 0.2 μL of 2 mM DCG-04. For inhibition assays, AF were first incubated together with 0.0, 0.1, 0.2, 0.3 or 0.4 μM purified NIa protein and then treated with 2 μM DCG-04 for 40 min in presence or absence of E-64. The reactions were stopped by the addition of 10 μL of 5× SDS-PAGE sample buffer. The resulting samples were analyzed via SDS-PAGE. Proteins attached to the nitrocellulose membranes were detected using HRP. To analyze the activity of TaRD21A and its altered proteins, the purified TaRD21A^R^-GST, TaRD21A^S^-GST and AF from wheat transgenic lines in the presence or absence of E-64 using 10 μM of the following substrates: Z-Phe-Arg-7-amido-4-methylcoumarin (AMC), Z-Arg-Arg-AMC (Sigma-Aldrich, Shanghai, China). The increase in fluorescence was continuously monitored for up to 10 min using a Cary Eclipse Fluorescence Spectrophotometer (Agilent, Santa Clara, USA)^18^. For in vitro cleavage assays, the sequence of PROWIP1 was amplified from a wheat cDNA library using specific primers (Supplementary Data 8). The putative cleavage sites within PROWIP1 were substituted with alanine in silico and then synthesized by staff at Sangon Biotech Incorporation (Shanghai, China) to produce mutant PROWIP1^M1^ and PROWIP1^M2^. PROWIP1, PROWIP1^M1^ and PROWIP1^M2^ were subsequently cloned individually into expressing vector which containing His tag. His-tagged PROWIP1, PROWIP1^M1^, and PROWIP1^M2^ were expressed and extracted from *E. coli* Rosetta (DE3) and then purified individually via Ni-NTA His-binding resin. Then, the purified PROWIP1-His, PROWIP1^M1^-His, PROWIP1^M2^-His or His empty vector (5 μM) was added to AF of *TaRD21A*^*R*^-OE, *TaRD21A*^*S*^-OE, *tard21a*-KO, YM158(S), Fielder(R) plants or purified TaRD21A-GST protein which was then incubated for 0-15 min. The resulting products were mixed with 10 μL 5×SDS-PAGE sample buffer for western blot assay using anti-His antibody. To perform a large-scale cleavage assay, purified PROWIP1-His, PROWIP1^M1^-His or PROWIP1^M2^-His (10 μM) was co-incubated with AF from the *TaRD21A*^*R*^-OE, *TaRD21A*^*S*^-OE, *tard21a*-KO, YM158(S) or Fielder(R) plants. The small peptides in the resultant product were enriched via 10 kDa Amicon centrifugation filters (EMD Millipore). The peptide fractions were subsequently infiltrated into the second leaf of YM158(S) at 2-week-old. At 6-hour post infiltration, the samples were harvested for western blot assay in which a phospho-p44/p42-specific antibody was used to detect the MAPK signals.

### MALDI TOF analysis

Extended Wip1 peptide (eWip1; PDEEKITRRSPLDEPIEWEKPKGRRPDIFPK, 2 mM) was digested using the recombinant TaRD21A^R^-GST and TaRD21A^S^-GST or GST for 2-h until the reaction stopped by addition of 1% TFA. Reactions were performed at pH 6.0 in 50 mM potassium phosphate buffer. 1.5 ml of the samples were mixed with an equal volume of the crystallization matrix (5 mg/ml a-cyano-4-hydroxy-trans-cinnamic acid in 50% acetonitrile, 0.1% TFA) on the MALDI target, and mass spectra were recorded with a AutoflexIII mass spectrometer (Bruker Daltonics) in the reflector mode with external calibration (Peptide Calibration Standard II; Bruker Daltonics). Flex Analysis 3.0 was used for data analysis with a mass tolerance of 50 ppm for ions.

### Statistical analysis

Microsoft Excel was used to determine the mean values and standard errors of the treatments. *T* test or Tukey’s test was performed with SPSS 16.0 software (SPSS, Inc., Chicago, IL) to determine the significance of differences. Significant differences with unequal variance between two treatments were determined with a probability (P) value.

### Reporting summary

Further information on research design is available in the Nature Portfolio Reporting Summary linked to this article.

### Supplementary information

Supplementary Information

Description of Additional Supplementary Files

Supplementary Data 1

Supplementary Data 2

Supplementary Data 3

Supplementary Data 4

Supplementary Data 5

Supplementary Data 6

Supplementary Data 7

Supplementary Data 8

Reporting Summary

### Source data

Source Data

## Data Availability

Data supporting the findings of this work are available within the paper and its Supplementary Information files. The raw RNA-seq data are available in the NCBI database under accessions PRJNA924088 and PRJNA1037474. Source data are provided with this paper.
