# Author Correction: A papain-like cysteine protease-released small signal peptide confers wheat resistance to wheat yellow mosaic virus

**DOI:** 10.1038/s41467-024-45406-9

**Published:** 2024-02-02

**Authors:** Peng Liu, Chaonan Shi, Shuang Liu, Jiajia Lei, Qisen Lu, Haichao Hu, Yan Ren, Ning Zhang, Congwei Sun, Lu Chen, Yaoyao Jiang, Lixiao Feng, Tianye Zhang, Kaili Zhong, Jiaqian Liu, Juan Zhang, Zhuo Zhang, Bingjian Sun, Jianping Chen, Yimiao Tang, Feng Chen, Jian Yang

**Affiliations:** 1 State Key Laboratory for Managing Biotic and Chemical Threats to the Quality and Safety of Agro-products, Key Laboratory of Biotechnology in Plant Protection of Ministry of Agriculture and Rural Affairs and Zhejiang Province, Institute of Plant Virology, Ningbo University, Ningbo, 315211 China; 2 National Key Laboratory of Wheat and Maize Crop Science/CIMMYT-China Wheat and Maize Joint Research Center/Agronomy College, Henan Agricultural University, Zhengzhou, 450002 China; 3 Hunan Plant Protection Institute, Hunan Academy of Agricultural Sciences, Changsha, 410152 China; 4 Institute of Hybrid Wheat, Beijing Academy of Agriculture and Forestry Sciences, Beijing, 100097 China

**Keywords:** Plant immunity, Agricultural genetics, Biotic, Viral pathogenesis

Correction to: *Nature Communications* 10.1038/s41467-023-43643-y, published online 27 November 2023

The original version of this article incorrectly stated the natural variant of TaRD21A as “glycine-to-threonine”. The correct version states “alanine-to-serine” in place of “glycine-to-threonine” in “Abstract”, and “alanine-to-serine change (Ser-96)” in place of “glycine-to-threonine change (Thr-96)” in the first paragraph of the second section of “Results” (under Fig. 1).

In addition, the original version of this article incorrectly stated the phosphorylation site of TaRD21A as “Thr-96”. The correct version states “serine” in place of “threonine” in “Abstract”, and “Ser-96” in place of “Thr-96” at the following locations throughout the article file: (1) the second last sentence of “Introduction”; (2) three locations under subheading “Phosphorylation of TaRD21A^R^ interferes with its interaction with Nia”; (3) first sentence under subheading “Natural variations in TaRD21A are responsible for subspecies-specific resistance to WYMV infection”; (4) title of Fig. 6; (5) legend of Fig. 6a; (6) the second last paragraph of “Discussion”; (7) the last paragraph of Discussion; and (8) toward the ending of the content under subheading “Transient expression in plants”.

These have been corrected in both the PDF and HTML versions of the article.

