# Supplementary material for: A papain-like cysteine protease-released small signal peptide confers wheat resistance to wheat yellow mosaic virus
Source: Nat Commun. 2023 Nov 27;14:7773. doi: 10.1038/s41467-023-43643-y (PMC10682394; doi:10.1038/s41467-023-43643-y)
Supplement: Supplementary file 1 — Supplementary Information [file 41467_2023_43643_MOESM1_ESM.pdf]

A papain-like cysteine protease-released small signal peptide confers wheat resistance to wheat yellow mosaic virus

Liu *et al.*

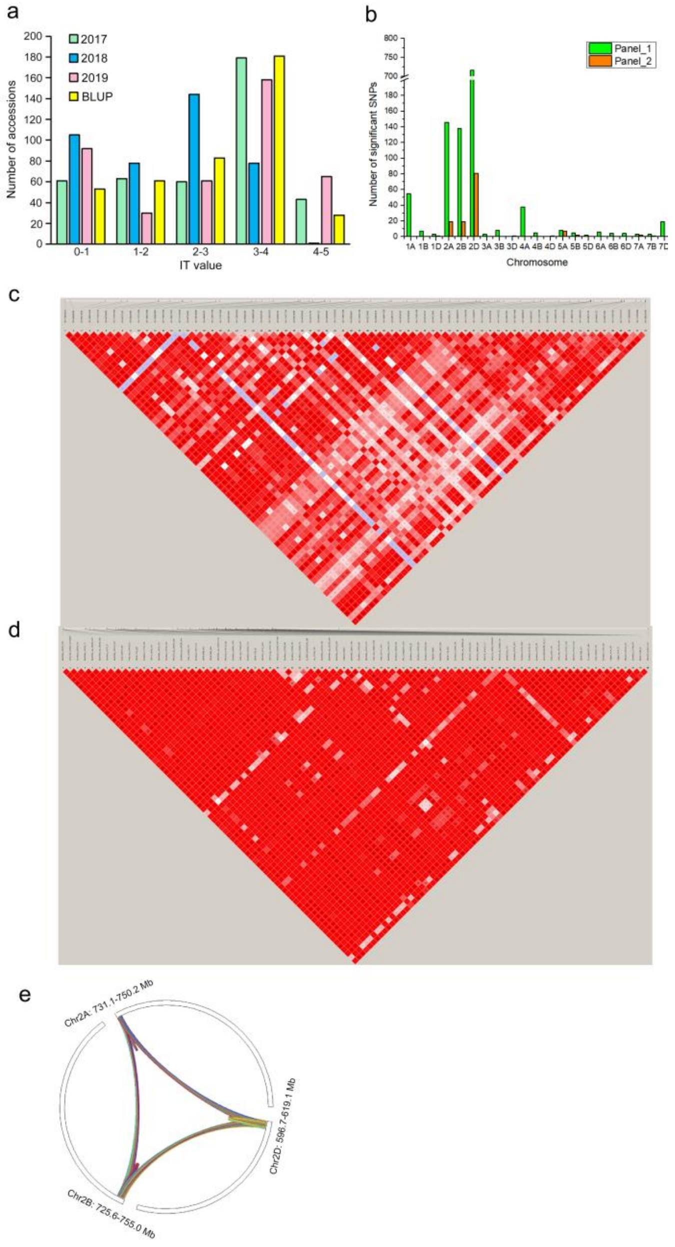

Supplementary Fig. 1. Phenotypic description and genome wide association study (GWAS) of wheat yellow mosaic (WYM) disease resistance. a. Distribution of Infection Type (IT) values among 406 wheat accessions screened for wheat yellow mosaic virus (WYMV) resistance in a disease nursery field during consecutive three years. BLUP, best linear unbiased prediction value. **b.** Distribution of significant SNPs among the wheat chromosomes in the two panels of wheat lines studied. **c.** Significant SNPs in the candidate region of 596.7-619.1 Mb on 2D in Panel I possessed a high linkage disequilibrium (LD). **d.** Significant SNPs in the candidate region of 596.9-613.3 Mb on 2D in Panel II possessed a high LD. **e**. Significant SNPs in the candidate region of 596.7-619.1 Mb on 2D in Panel I possessed a high linkage disequilibrium (LD). **e.** Collinearity analysis of candidate intervals for WYMV resistance located on 2A (731.1-750.2 Mb), 2B (725.9-755.0 Mb), and 2D (596.9-619.1 Mb). Source data are provided as a Source Data file.

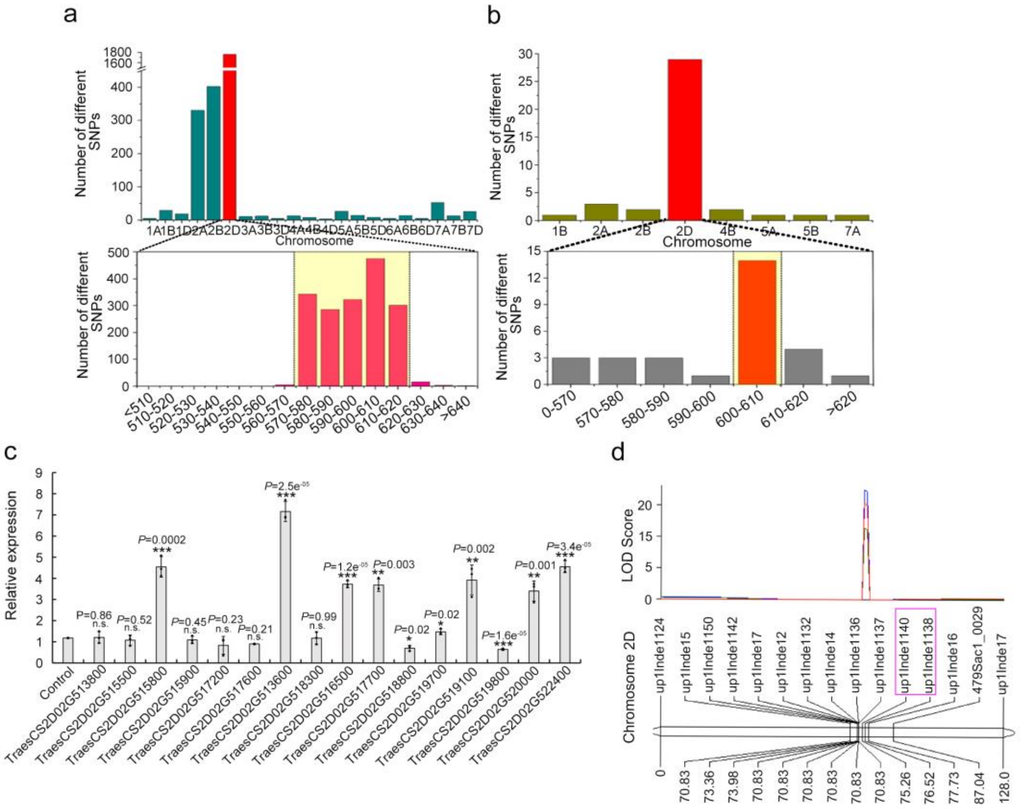

Supplementary Fig. 2. Localization of two bi-parental genetic populations (UP-RIL and BJ-DH) and screening of candidate genes. a and b. Results of bulked segregant analysis (BSA) based on wheat 660K SNP array (BSA-660K) in UP-RIL population (a) and wheat 55K SNP array (BSA-55K) in BJ-DH population (b). **c.** Transcript profiles of selected candidate genes in resistant cultivar Jingshuang 16 under WYMV infection. The leaves of Jingshuang 16 inoculated with WYMV and then sampled at 14 days post inoculation (dpi). The leaves inoculated with FES buffer were used as control. Asterisks indicate significant differences between Control and WYMV treated plants. Values of qRT-PCR is the mean ± SD (two-sided *t* test, n=3 biologically independent experiments, *** $P<0.001$, ** $P<0.01$, * $P<0.05$, n.s., no significantly differences). **d.** LOD contours for quantitative trait loci (QTL) to WYMV resistance on 2D identified by inclusive composite interval mapping (ICIM) in the Bainong64/Jingshuang16 (BJ) population. Source data are provided as a Source Data file.

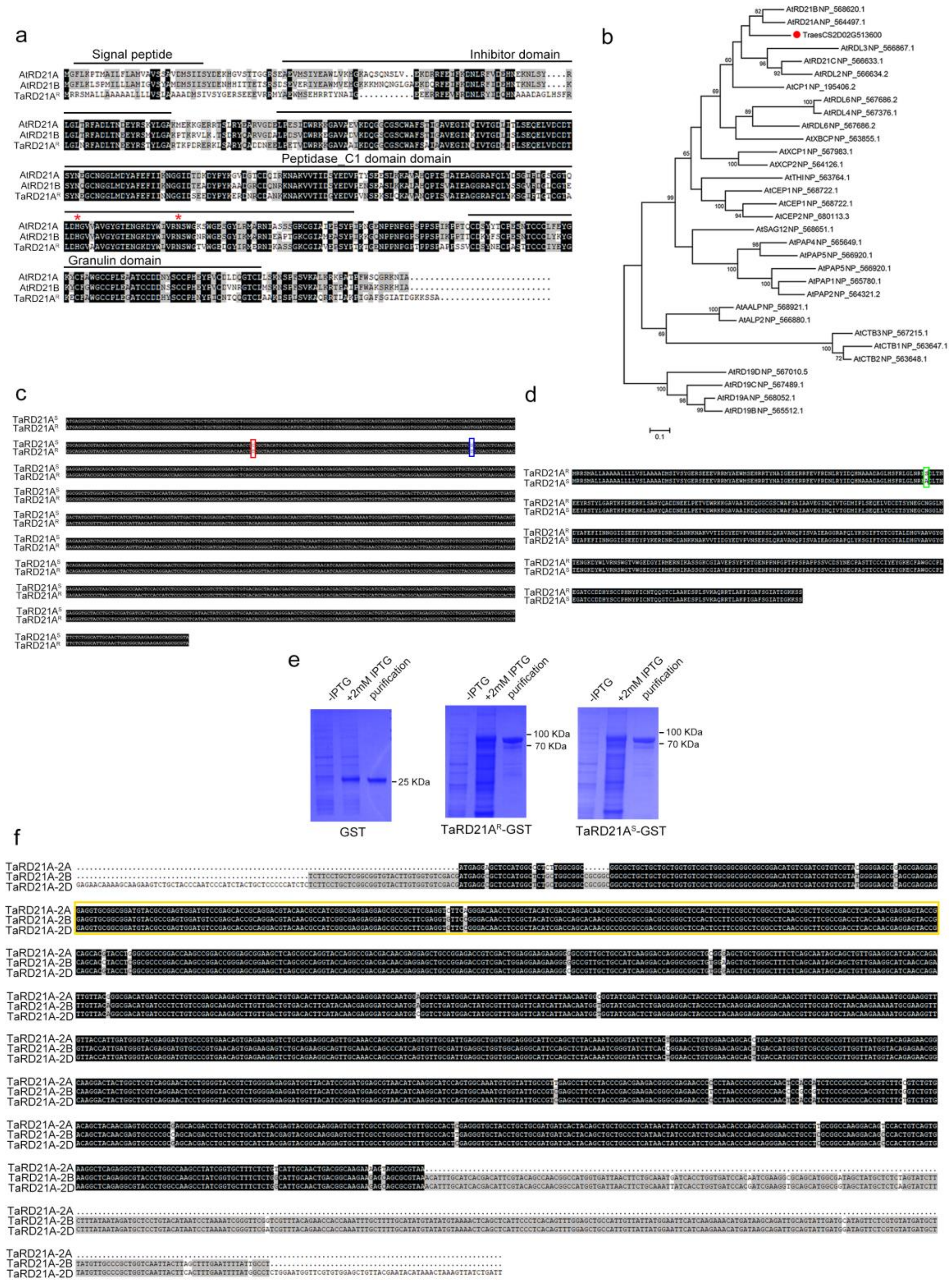

Supplementary Fig. 3. Sequence analysis of TaRD21A^R. a. Multiple sequence alignment of TaRD21A^R, AtRD21A and AtRD21B. Identical amino acid residues are highlighted in black, and similar residues are highlighted in gray. The red asterisks indicate the conserved catalytic triad Cys-His-Asn. **b.** A maximum-likelihood phylogenetic tree constructed using sequences of *Arabidopsis* PLCPs proteins and TraesCS2D02G513600 after multiple sequence alignments. The numbers at the

branches indicate the bootstrap values estimated using 500 replications. **c.** The coding sequencing of *TaRD21A*^{R} and *TaRD21A*^{S} was aligned by DNAMAN software. *TaRD21A* in the resistant cultivars was designated as *TaRD21A*^{R} and that in the susceptible cultivars was designated as *TaRD21A*^{S}, respectively. Red box: a synonymous nucleotide polymorphism. blue box: a non-synonymous nucleotide polymorphism. **d.** Multi-sequence alignment of TaRD21A^R and TaRD21A^S. Green box: amino acid change from Glycine in susceptible cultivars to threonine in resistant cultivars. **e.** TaRD21A^R-GST, TaRD21A^S-GST or GST was expressed and purified from *E. coli* via GST-binding resin respectively for SDS gel staining. **f.** Multiple sequence alignment of the coding sequences for the three TaRD21A copies. Yellow box: The fragments for qRT-PCR assay. Source data are provided as a Source Data file.

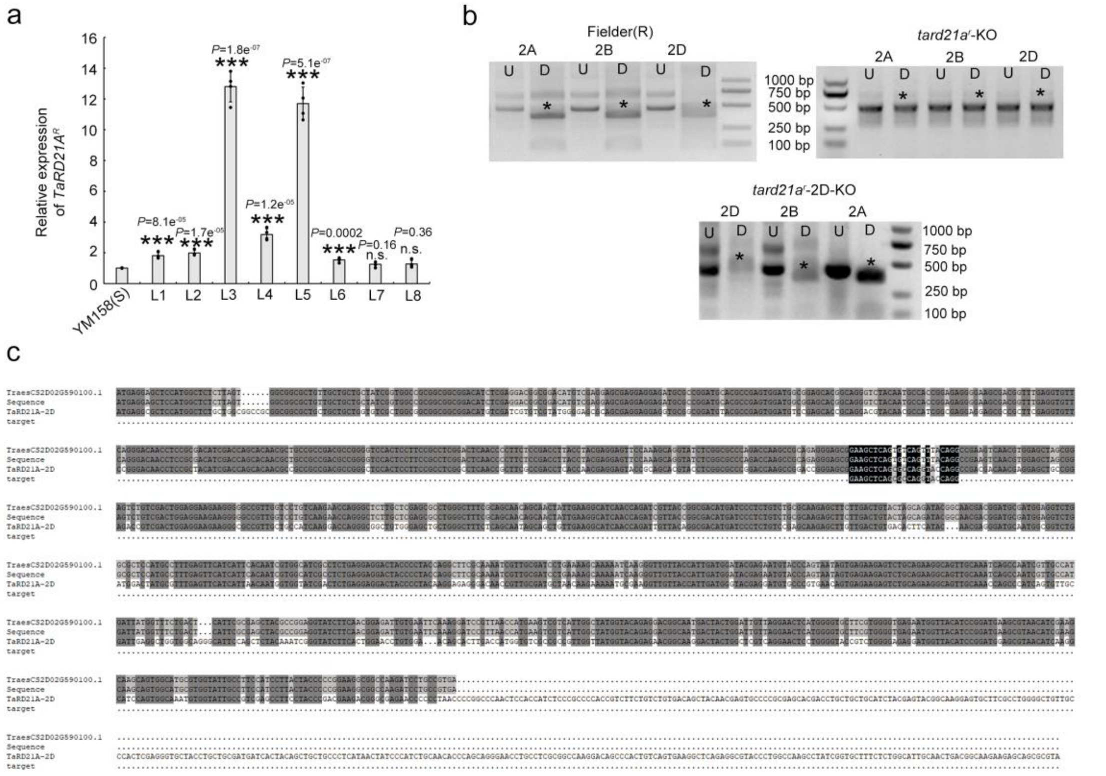

Supplementary Fig. 4. Positive lines selection of *TaRD21A*^{R}-OE, *tard21a*^{r}-KO and *tard21a*^{r}-2D-KO. a. The expression levels of *TaRD21A*^{R} in the leaves of 8 positive transgenic plants in T_0 generation and YM158(S) as detected by qRT-PCR. Asterisks indicate significant differences between each treatment. Values of qRT-PCR is the mean ± SD (two-sided *t* test, n=4 biologically independent experiments, ***P<0.001, n.s., no significant). **b**. PCR/RE assay was used to detect the homozygous in *tard21a*^{r}-KO and *tard21a*^{r}-2D-KO. genomic DNA was extracted from *tard21a*^{r}-KO and *tard21a*^{r}-2D-KO for amplification of specific fragment in *TaRD21A-2A*, *TaRD21A-2B*, and *TaRD21A-2D* surrounding gRNA target site. The resulting amplicons including the gRNA target site were digested by *Kpn*I restriction enzymes. The DNA in Fielder(R) was used as control. Asterisks indicate the digestion production. **c.** The editing construct is not also targeting other gene. Source data are provided as a Source Data file.

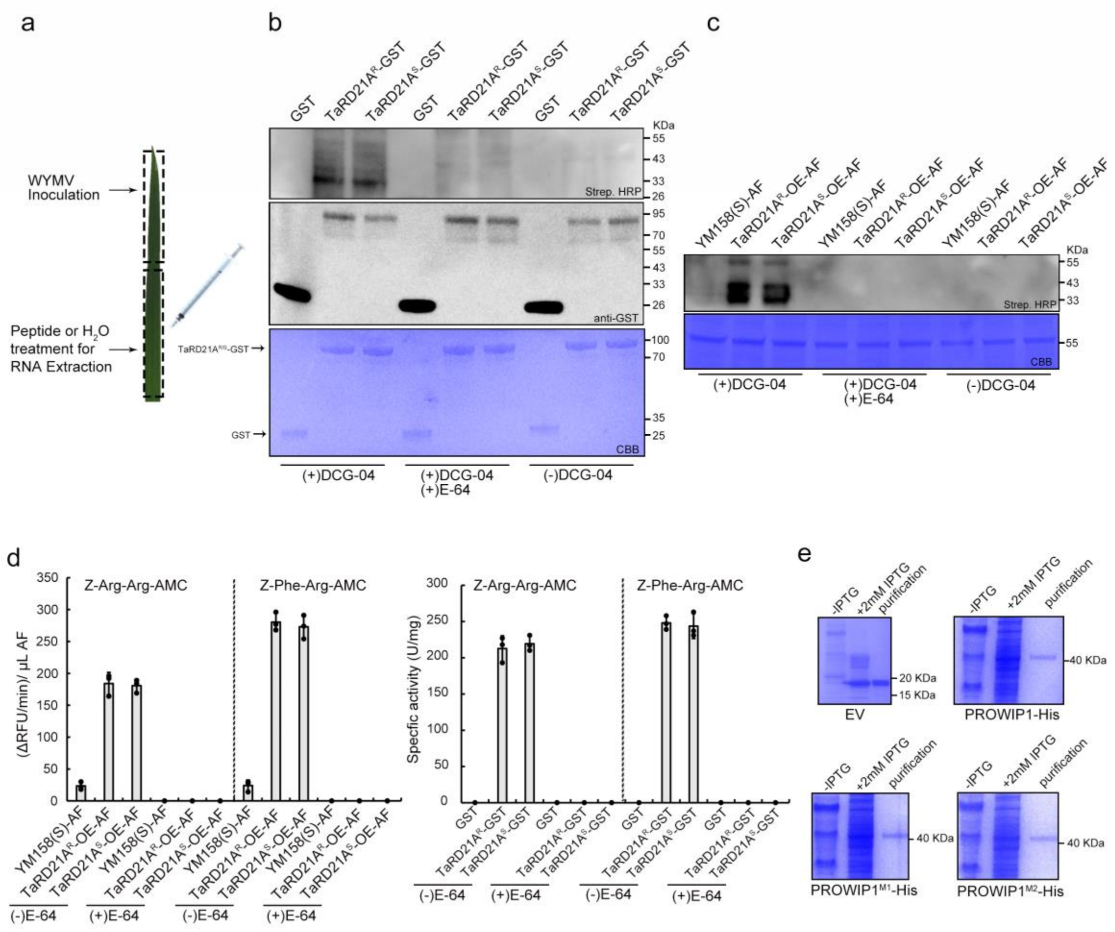

Supplementary Fig. 5. TaRD21A^{R}-mediated release of Wip1 confers wheat resistance to WYMV infection in the leaves of YM158(S). a. Schematic illustration of wheat leaf treatment and sample preparation for subsequent qRT-PCR analyses**.** At 6 hours post treatment (hpt) of peptides, Peptide fractions or H_2O, the leaves were inoculated with WYMV. **b and c.** TaRD21A^{R}-GST, TaRD21A^{S}-GST and GST was expressed and purified from *E. coli* (BL21). The AF were prepared fromYM158(S), *TaRD21A^{R}*-OE and *TaRD21A^{S}*-OE line leaves. The AF and GST fusion protein was labeled DCG-04 in the presence or absence of E-64. (-)DCG-04 indicated that the AF and GST fusion protein without DCG-04 was used for distinguish from background signals. Coomassie Blue staining shows the assayed proteins loaded. **d.** Protease activity assay withdifferent substrates. Protease activity in AF of YM158(S), *TaRD21A^{R}*-OE, *TaRD21A^{S}*-OE line leaves, TaRD21A^{R}-GST, TaRD21A^{S}-GST and GST was monitored with Z-Phe-Arg-AMC and Z-Arg-Arg-AMC substrates in the presence and absence of E-64. Three times each experiment was repeated independently. **e.** PROWIP1-His, PROWIP1^{M1}-His, PROWIP1^{M2}-His and expression vector containing His tag (EV) was expressed and purified from *E. coli* via Ni-NTA His-binding resin respectively for SDS gel staining. The data in **b** and **c** are representative of n=3 independent experiments. Source data are provided as a Source Data file.

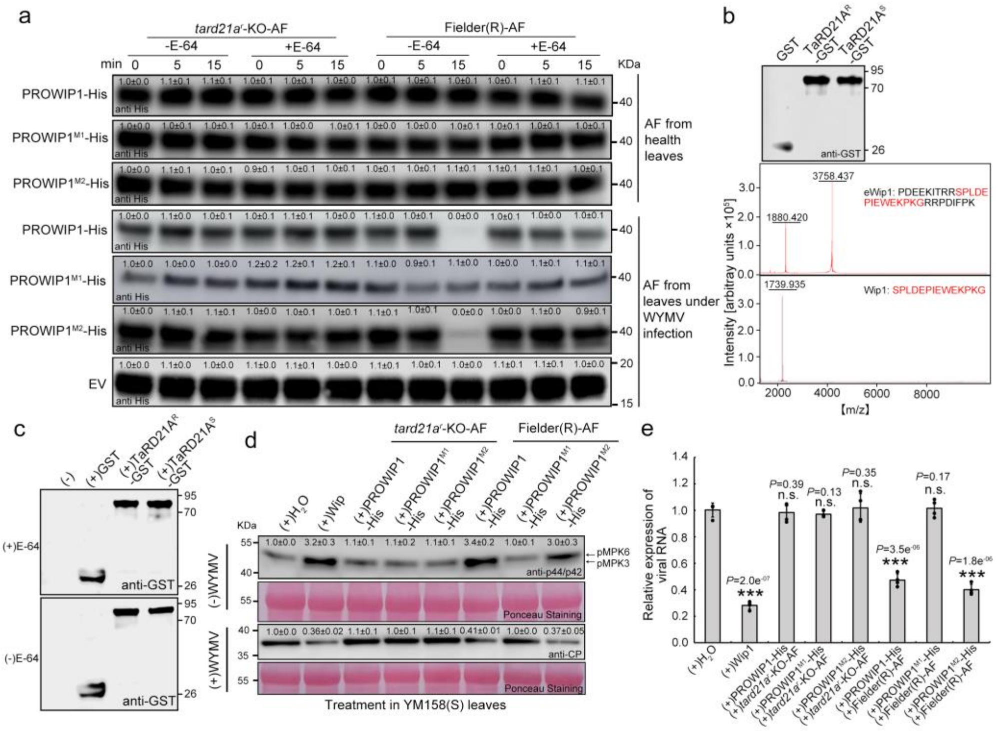

Supplementary Fig. 6. TaRD21A^{R}-mediated release of Wip1 confers wheat resistance to WYMV infection. a. *In vitro* cleavage assays using PROWIP1, PROWIP1^{M1} and PROWIP1^{M2}-His as substrates, respectively. PROWIP1-His, PROWIP1^{M1}-His, and PROWIP1^{M2}-His were individually incubated with AF from the leaves of *tard21a^{r}*-KO or Fielder(R) with or without WYMV infection. Western blot assays were performed using an anti-His antibody. EV is representative of expressing vector which containing His tag and used for fused PROWIP1 or its altered protein with His tag. **b.** Western blot analysis of purified TaRD21A^{R}-GST, TaRD21A^{S}-GST and GST which were used in Fig 4e. Masses of eCLEL6 (PDEEKITRRSPLDEPIEWEKPKGRRPDIFPK) and mature CLEL6 (DYPQPHRKPPIHNE) are indicated by LC/MS analysis. **c.** Western blot analysis of purified TaRD21A^{R}-GST, TaRD21A^{S}-GST and GST which were used in Fig 4f. **d and e.** PROWIP1-His, PROWIP1^{M1}-His, and PROWIP1^{M2}-His were individually incubated with AF from *tard21a^{r}*-KO or Fielder(R) leaves with WYMV infection. The small peptides were enriched in the production of PROWIP1: His, PROWIP1^{M1}: His or PROWIP1^{M2}: His treated with active proteases using 10 kDa Amicon centrifugation filters (EMD Millipore). Each Peptide fractions from the co-incubated samples were individually infiltrated into the leaves of YM158(S). 6 hours later, the infiltrated wheat leaves were sampled for analysis of MAPK signals or inoculated with WYMV for testing the accumulation of viral protein. The wheat leaves treated with H_2O were used as negative control. The accumulation of viral RNA in the infiltrated leaves was confirmed by qRT-PCR assay at 7 dpi. Asterisks indicate significant differences between each treatment. Values of qRT-PCR is the mean ± SD (two-sided *t* test, n=4 biologically independent experiments, ***P<0.001, n.s., no significant). The data in **a-d** are representative of n=3 independent experiments. Source data are provided as a Source Data file.

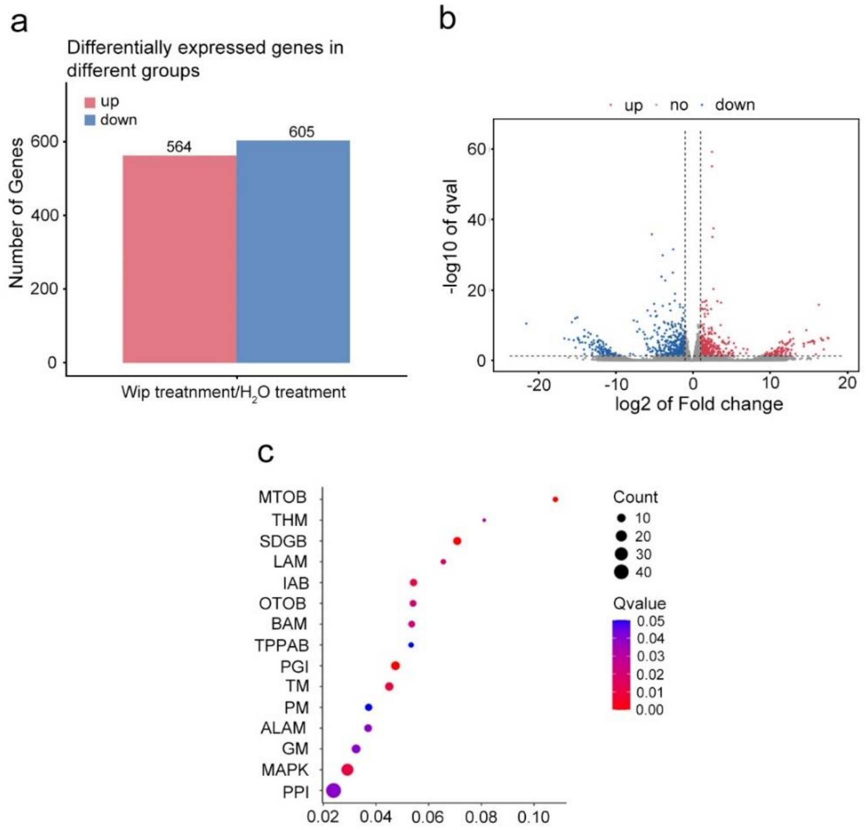

Supplementary Fig. 7. The transcriptome analyses in the leaves of YM158(S) treated with Wip1 using transcriptome analyses. a and b. The numbers of differentially expressed genes in wheat leaves treated with Wip1. **c.** KEGG analysis of differentially regulated genes identified through RNA-seq using wheat leaf samples at 6 hours post treatment with Wip1 or H_2O. Only differentially regulated genes (above or below a logFC threshold of ~± 1) were selected for this analysis. MTOB: Mannose type O-glycan biosynthesis; THM: Taurine and hypotaurine metabolism; SDGB: Stilbenoid, diarylheptanoid and gingerol biosynthesise; LAM: Linoleic acid metabolism; IAB: Indole alkaloid biosynthesis; OTOB: Other types of O-glycan biosynthesis; BAM: beta-Alanine metabolism; TPPAB: Tropane, piperidine and pyridine alkaloid biosynthesis; PGI: Pentose and glucuronate interconversions; TM: Tryptophan metabolism; PM: Phenylalanine metabolism; ALAM: alpha-Linolenic acid metabolism; GM: Glycerolipid metabolism; MAPK: MAPK signaling pathway-plant; PPI: Plant-pathogen interaction.

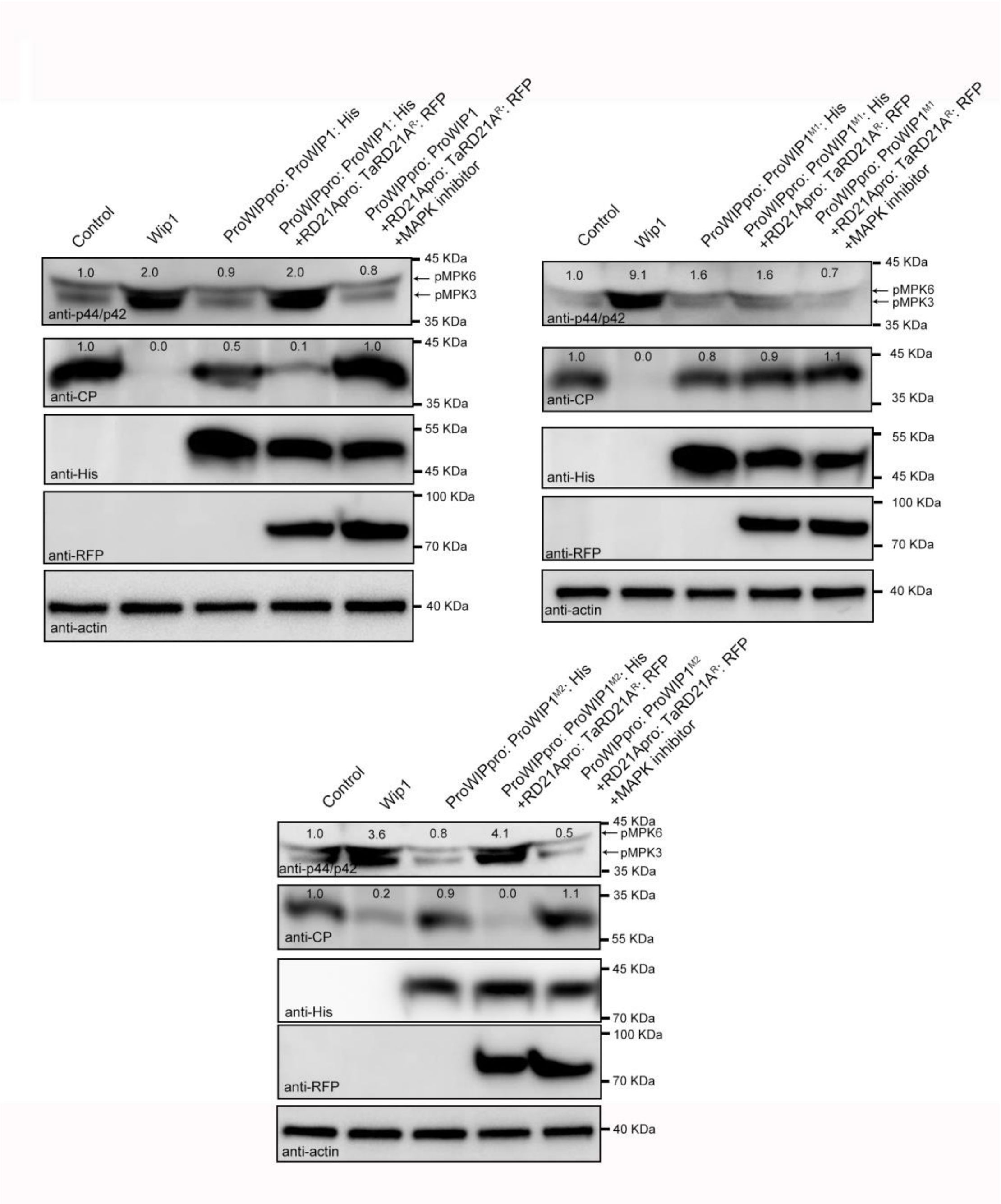

Supplementary Fig. 8. PROWIP1 and its altered protein is involved in MAPK signal and antiviral response to WYMV infection. PROWIP1, PROWIP1^{M1} and PROWIP1^{M2} fused to His tag was expressed under the control of its native promoter (PROWIPpro) and then co-expressed with RD21Apro: TaRD21A^{R}: RFP followed by inoculated with WYMV. Samples treated with H_2O or Wip1 were used as negative or positive control. U0126 was used to blocks the MAPK signal transduction. The data in are representative of n=3 independent experiments. Source data are provided as a Source Data file.

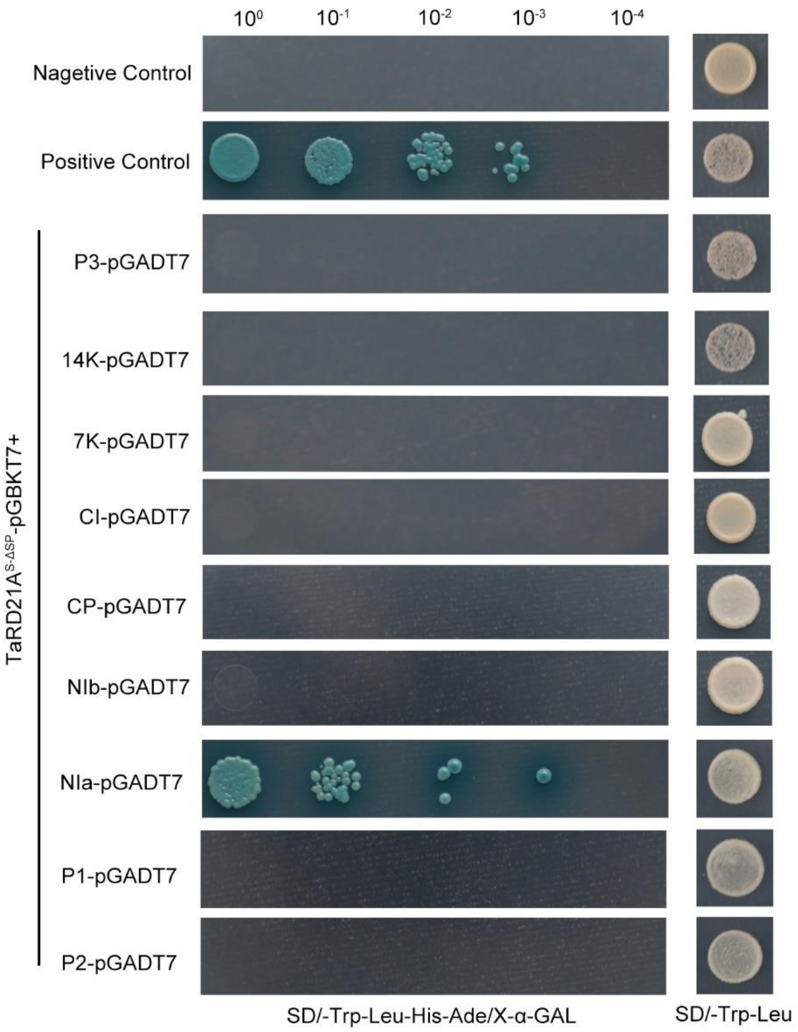

Supplementary Fig. 9. TaRD21A^{S} without signal peptide (TaRD21A$^{S\text{-}\Delta SP}$) interacted with WYMV-encoded protein NIa in a yeast two-hybrid (Y2H) assay. AH109 strain cultures containing TaRD21A$^{S\text{-}\Delta SP}$ in the GAL4 DNA-binding domain (BD) and WYMV coding proteins in the GAL4 activation domain (AD) on selective medium. Positive control, the interaction between SV40 large T-antigen (T) and murine p53 (53) T-AD+53-BD. Negative control, the interaction between SV40 large T-antigen (T) and human lamin C (Lam), T-AD+Lam-BD.

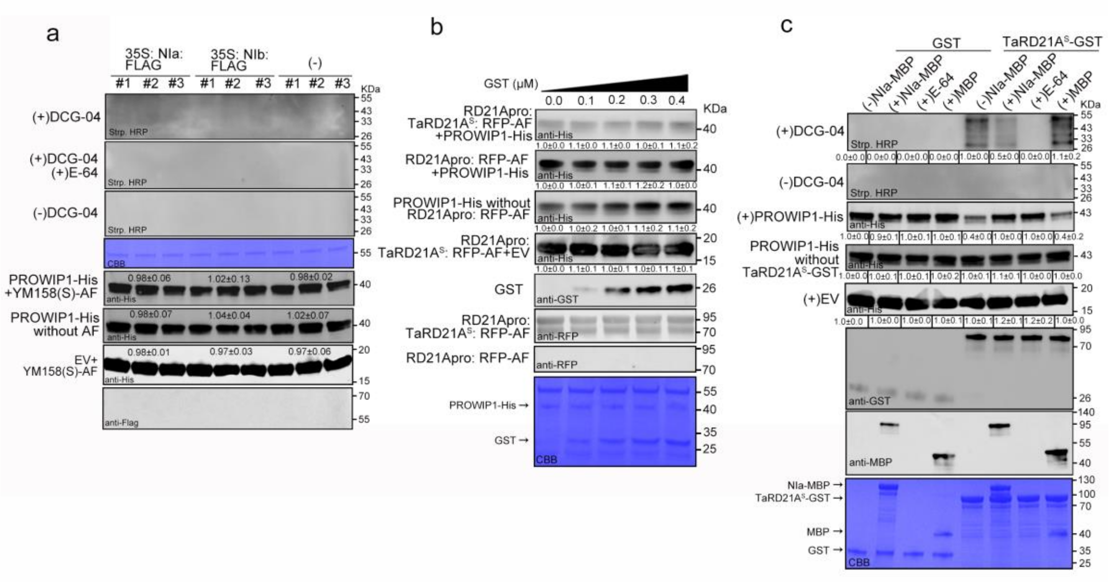

Supplementary Fig. 10. The control for Fig. 5. a. The effect of NIa on protein activity in the AF from the leaves of YM158(S). 35S: NIa: FLAG was transiently expressed in the leaves of YM158(S) by particle bombardment-mediated transformation. AF from these assayed leaves was then incubated with DCG-04 in the presence or absence of E-64. The resulting samples were analyzed by western blot assays using a Streptavidin-HRP conjugate. PROWIP1 or EV was used as substrate in the *in vitro* cleavage assays. The AF from wheat leaves with or without the expression of NIb were used as control. **b.** Effect of GST on the cysteine proteases activity of TaRD21A^S in the AF from *N. benthamiana* leave expressing RD21Apro: TaRD21A^S: RFP or RD21Apro: RFP, which was determined by western blot assay. **c**. Effect of NIa on the cysteine proteases activity of TaRD21A^S-GST or GST using DCG-04 labelling. The recombinant PROWIP1-His was incubated with purified TaRD21^S-GST and GST for 80 min respectively in the presence or absence of NIa-MBP or MBP. Cleavage was detected by western blot assay using His antibody. EV is representative of expressing vector which containing His tag and used for fused PROWIP1 or its altered protein with His tag. The data are representative of n=3 independent experiments. The coomassie blue staining shows the assayed protein loaded. Source data are provided as a Source Data file.

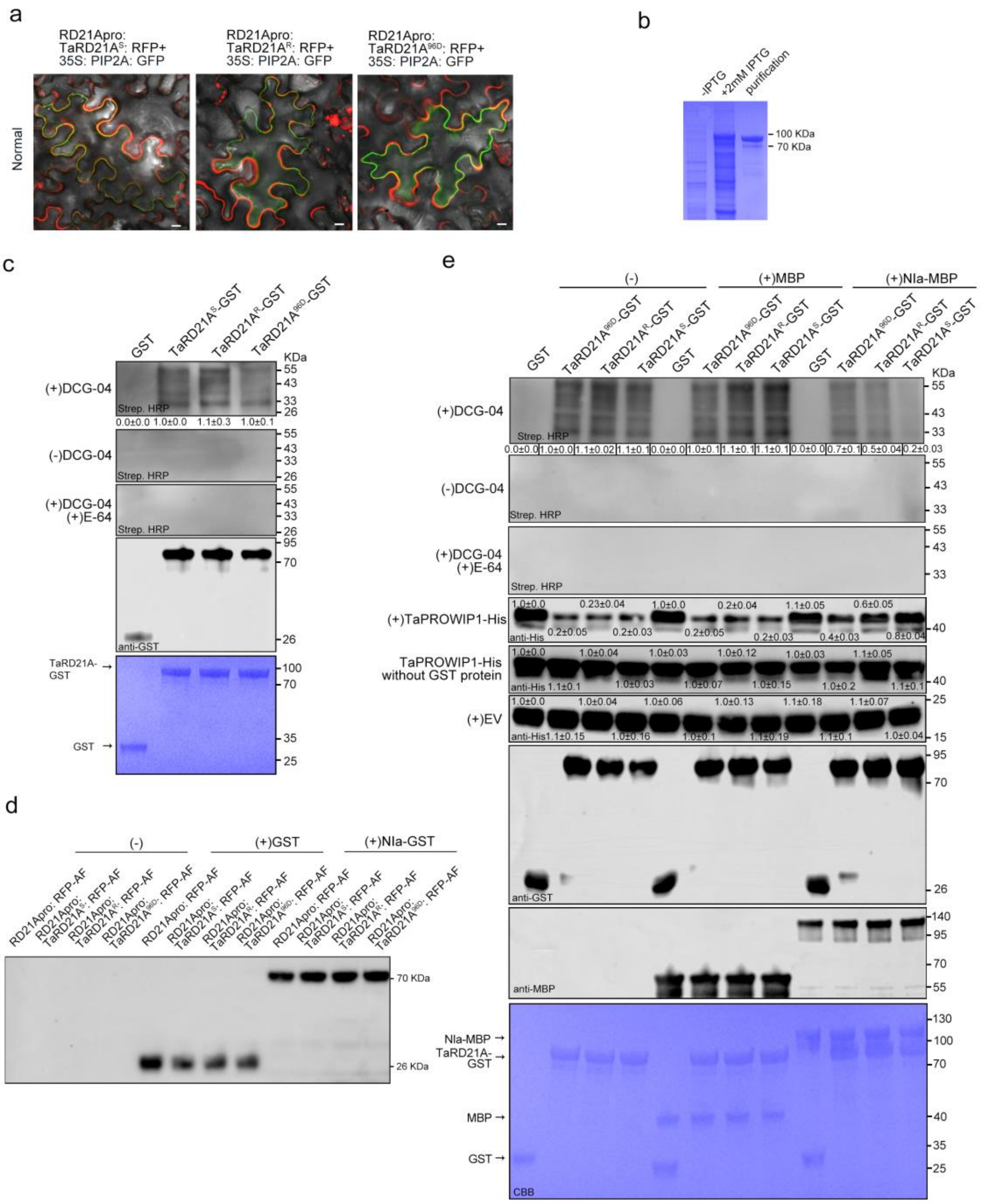

Supplementary Fig. 11. the phosphorylation of TaRD21A impacts the inhibition of TaRD21A proteinase activity by NIa *in vitro*. a. Subcellular localization of RD21Apro: TaRD21A^{S}: RFP, RD21Apro: TaRD21A^{R}: RFP, and RD21Apro: TaRD21A^{96D} in *N. benthamiana* leaf cells as shown by confocal microscopy. RD21Apro: TaRD21A^{S}: RFP, RD21Apro: TaRD21A^{R}: RFP, and RD21Apro: TaRD21A^{96D} :RFP was co-expressed with 35S: PIP2A: GFP in in *N. benthamiana* leaves. Bar, 10 µm. **b.** TaRD21A^{96D}-GST, was expressed and purified from *E. coli* via GST-binding resin respectively for SDS gel staining. **c.** TaRD21A^{S}-GST, TaRD21A^{R}-GST or TaRD21A^{96D}-GST were abeled with DCG-04, followed by western blot assay for determining the activity. (-)DCG-04 indicated that GST fusion protein without a non-probe control was used for distinguish from background signals. GST was used as

negative control. **d.** Western blot analysis of NIa-GST and GST which were used in Fig 6h. **e.** TaRD21A^{S}-GST, TaRD21A^{R}-GST, TaRD21A^{96D}-GST and GST was labeled with DCG-04, in the presence NIa-MBP or MBP. TaRD21A activity was determined by western blot assay using streptavidin-HRP conjugate and PROWIP1-His fusion was used as the substrate. EV is representative of expressing vector which containing His tag and used for fused PROWIP1 or its altered protein with His tag. The data in **c-e** are representative of n=3 independent experiments. The coomassie blue staining in **c** and **e** shows the assayed proteins loaded. Source data are provided as a Source Data file.

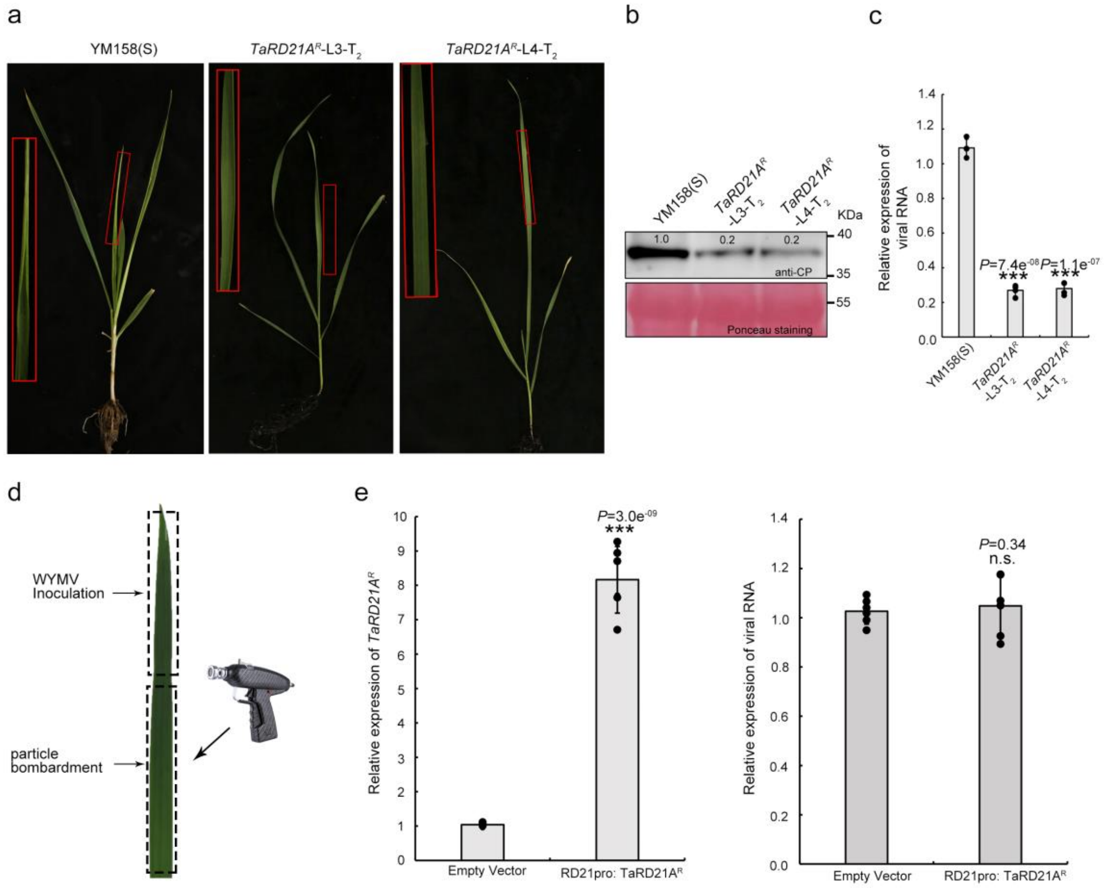

Supplementary Fig. 12. Thr-96 of TaRD21A^R is involved in the wheat resistance to WYMV infection. a. The phenotype of *TaRD21A^R*-T_2-L3 and *TaRD21A^R*-T_2-L4 inoculated with WYMV at 40 dpi. **b and c**. Detection of viral RNA and protein in the assayed wheat plants by qRT-PCR and western blot assays, respectively. Values of qRT-PCR are the mean ± SD (Tukey's test, n=4 biologically independent experiments, ****P*<0.001, n.s., no significant). **d.** Schematic illustration of particle bombardment assay and sample preparation for subsequent qRT-PCR analyses**.** the leaves of the resistant cultivar UC1110 was used for particle bombardment. After 6 hours post transiently expressing RD21Apro: TaRD21A^R, the leaves were inoculated with WYMV. **e.** the expression levels of *TaRD21A* and viral RNA in RD21Apro: TaRD21A^R transiently expression leaves in assayed wheat leaves was determined by qRT-PCR assay. The leaves of UC1110 transiently expressed empty vector was used as control. Asterisks indicate significant differences between each treatment. Values of qRT-PCR is the mean ± SD (two-sided *t* test, n=6 biologically independent experiments, ****P*<0.001, n.s., no significant). The data in **b** are representative of n=3 independent experiments. Source data are provided as a Source Data file.

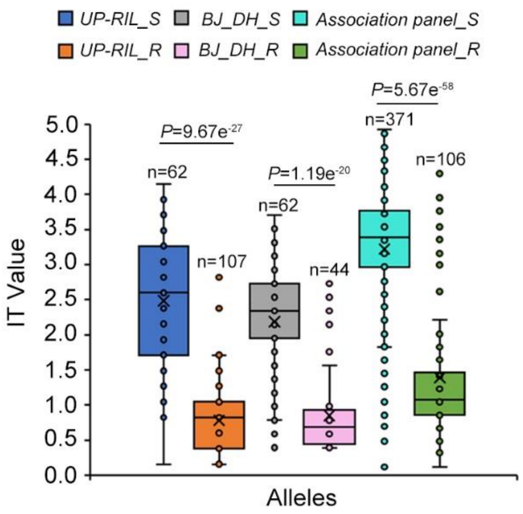

Supplementary Fig. 13. Statistical analysis of WYMV resistance in materials with resistant alleles and susceptible alleles in two bi-parental genetic populations (UP-RIL and BJ-DH) and two associated populations (406 wheat accessions). n, represent the number of wheat accessions with the corresponding alleles. Statistics: for both datasets, two-sided *t* test was performed. For box-plot, the horizontal lines from top to bottom represent the maximum, first quartile, median, third quartile, and minimum of the total data, respectively. The cross in the middle of the box represents the average. Source data are provided as a Source Data file.

a

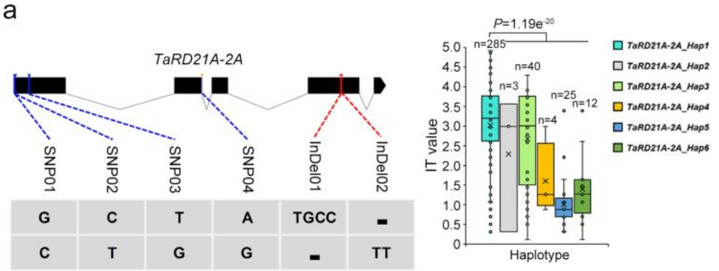

Haplotype	SNP01	SNP02	SNP03	SNP04	InDel01	InDel02	IT value for WYMV resistance	Number of varieties
TaRD21A-2A_Hap1	G	C	T	A	+	-	3.02	285
TaRD21A-2A_Hap2	G	C	T	A	-	+	1.60	4
TaRD21A-2A_Hap3	G	C	T	G	+	-	1.01	25
TaRD21A-2A_Hap4	G	C	G	A	+	-	2.29	3
TaRD21A-2A_Hap5	C	T	G	A	+	-	2.68	40
TaRD21A-2A_Hap6	C	T	G	G	+	-	1.40	12
Variation type	Ser3Thr	Ala6Val	Ser46Ala	Splice	Frameshift	Frameshift		

b

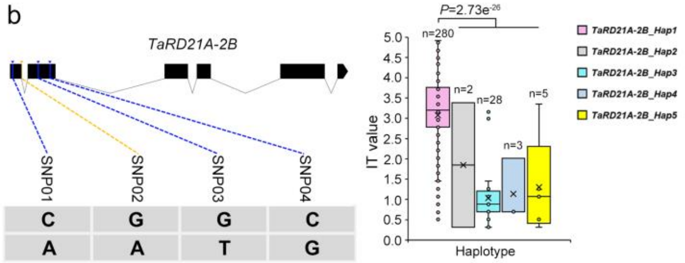

Haplotype	SNP01	SNP02	SNP03	SNP04	IT value for WYMV resistance	Number of varieties
TaRD21A-2B_Hap1	C	G	G	C	3.07	280
TaRD21A-2B_Hap2	C	G	G	G	1.85	2
TaRD21A-2B_Hap3	C	A	G	C	1.03	28
TaRD21A-2B_Hap4	C	A	T	C	1.14	3
TaRD21A-2B_Hap5	A	G	G	C	1.30	5
Variation type	Ala9Glu	Splice	Ala96Ser	Thr135Ser		

c

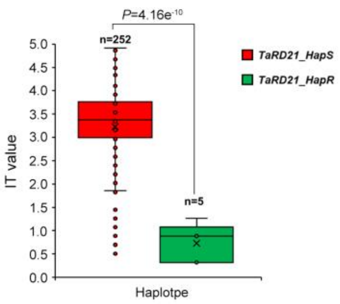

Supplementary Fig. 14. Haplotype analysis of *TaD21A-2A* and *TaD21A-2B* in natural varieties. a and b. Polymorphic distribution of the *TaRD21A-2A* and *TaRD21A-2B* coding regions. n, represent the number of wheat accessions with the corresponding haplotype. Statistics: for both datasets, two-sided *t* test was performed. **c.** The combination of superior haplotypes for three homoeologous copies of *TaRD21A*. For box-plot in **a-c**, the horizontal lines from top to bottom represent the maximum, first quartile, median, third quartile, and minimum of the total data, respectively. The cross in the middle of the box represents the average. Source data are provided as a Source Data file.

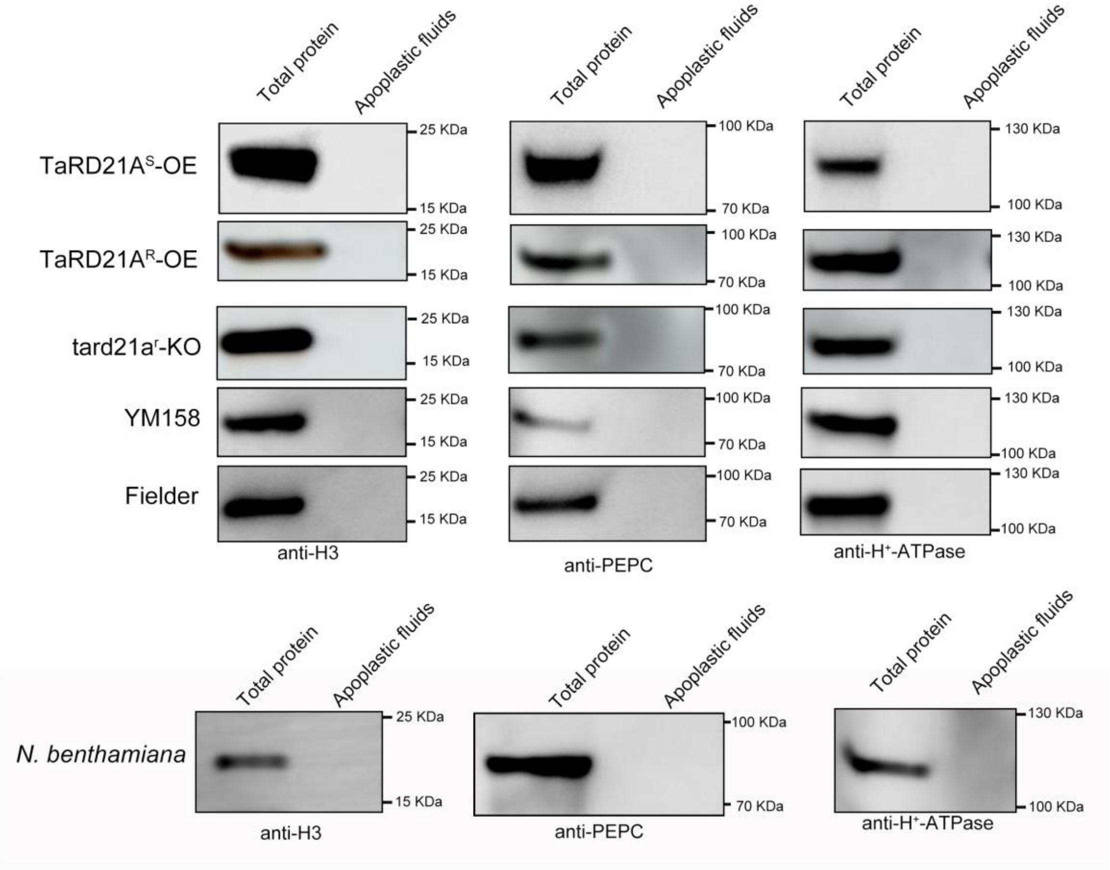

Supplementary Fig. 15. The AF was verified the absence of nuclear, cytoplasmic, or plasma membrane components. H3, PEPC and H^+-ATPase was used to indicate the components of nuclear, cytoplasmic, or plasma membrane. Detection of H3, PECP and H^+-ATPase in the extracted AF from wheat or *N. benthamiana* leaves by western blot assay using the H3, PECP and H^+-ATPase antibody. Source data are provided as a Source Data file.
