# Supplementary material for: A papain-like cysteine protease-released small signal peptide confers wheat resistance to wheat yellow mosaic virus
Source: Nat Commun. 2023 Nov 27;14:7773. doi: 10.1038/s41467-023-43643-y (PMC10682394; doi:10.1038/s41467-023-43643-y)
Supplement: Supplementary file 2 — Description of Additional Supplementary Files [file 41467_2023_43643_MOESM2_ESM.pdf]

Description of Additional Supplementary Files

File Name: Supplementary Data 1
Description: **The list of total significant SNPs detected on 2D in two panels**. Note: Two sides and the multiple test correction is conducted with Bonferroni method.

File Name: Supplementary Data 2
Description: **The list of total different SNPs detected on 2D in the UP-RIL populations based on Wheat 660K SNP array.**

File Name: Supplementary Data 3
Description: **The list of total different SNPs detected on 2D in the BJ-DH populations based on Wheat 55K SNP array.**

File Name: Supplementary Data 4
Description: **QTL for WYMV resistance identified in UC1110/PI610750 (UP-RIL) and Bainong64/Jingshuang16 (BJ-DH) genetic linkage populations.**

File Name: Supplementary Data 5
Description: **Gene list of 23 different expression gene that identifeied by bulked segregant transcriptome using RNA-seq (BSR-Seq).**

File Name: Supplementary Data 6
Description: **Gene list possessed nonsynonymous variation between UC1110 and PI610750 that identifeied by genome resequencing.**

File Name: Supplementary Data 7
Description: **indentification of immune signaling peptides in leaf apoplastic fluids from TaRD21A-OE transgenic**

File Name: Supplementary Data 8
Description: **the primer used in this study**
