# Supplementary material for: A papain-like cysteine protease-released small signal peptide confers wheat resistance to wheat yellow mosaic virus
Source: Nat Commun. 2023 Nov 27;14:7773. doi: 10.1038/s41467-023-43643-y (PMC10682394; doi:10.1038/s41467-023-43643-y)
Supplement: Supplementary file 11 — Reporting Summary [file 41467_2023_43643_MOESM11_ESM.pdf]

nature portfolio

Corresponding author(s): Jianping Chen, Yimiao Tang, Feng Chen and Jian Yang

Last updated by author(s): Nov 3, 2023

Reporting Summary

Nature Portfolio wishes to improve the reproducibility of the work that we publish. This form provides structure for consistency and transparency in reporting. For further information on Nature Portfolio policies, see our Editorial Policies and the Editorial Policy Checklist.

Statistics

For all statistical analyses, confirm that the following items are present in the figure legend, table legend, main text, or Methods section.

n/a	Confirmed	
☐	☒	The exact sample size (*n*) for each experimental group/condition, given as a discrete number and unit of measurement
☐	☒	A statement on whether measurements were taken from distinct samples or whether the same sample was measured repeatedly
☐	☒	The statistical test(s) used AND whether they are one- or two-sided<br>*Only common tests should be described solely by name; describe more complex techniques in the Methods section.*
☒	☐	A description of all covariates tested
☒	☐	A description of any assumptions or corrections, such as tests of normality and adjustment for multiple comparisons
☐	☒	A full description of the statistical parameters including central tendency (e.g. means) or other basic estimates (e.g. regression coefficient) AND variation (e.g. standard deviation) or associated estimates of uncertainty (e.g. confidence intervals)
☒	☐	For null hypothesis testing, the test statistic (e.g. *F*, *t*, *r*) with confidence intervals, effect sizes, degrees of freedom and *P* value noted<br>*Give P values as exact values whenever suitable.*
☒	☐	For Bayesian analysis, information on the choice of priors and Markov chain Monte Carlo settings
☒	☐	For hierarchical and complex designs, identification of the appropriate level for tests and full reporting of outcomes
☒	☐	Estimates of effect sizes (e.g. Cohen's *d*, Pearson's *r*), indicating how they were calculated

Our web collection on statistics for biologists contains articles on many of the points above.

Software and code

Policy information about availability of computer code

Data collection	Nanotemper NT.115<br>Confocal microscopic images were aquired with a TCS SP8 X confocal microscopy.<br>ThermoFisher QuantStudio 5 for qPCR data collection and analysis.
Data analysis	1. GWAS analysis was implemented using GAPIT Version 3 packages in R software based on the mixed linear model (PCA+K)<br>2. QTL IciMapping V4.1 (https://isbreeding.caas.cn/rj/qtllcmapping/294444.htm).<br>3.Mo.Affinity Analysis v2.3<br>4.MEGA 7.0 software<br>5.Microsoft excel.<br>6.GraphPad Prism 8.<br>7.MO.Affinity Analysis v2.3<br>8. SPSS 16.0 software<br>9 .Image J

For manuscripts utilizing custom algorithms or software that are central to the research but not yet described in published literature, software must be made available to editors and reviewers. We strongly encourage code deposition in a community repository (e.g. GitHub). See the Nature Portfolio guidelines for submitting code & software for further information.

Data

Policy information about availability of data

All manuscripts must include a data availability statement. This statement should provide the following information, where applicable:

- Accession codes, unique identifiers, or web links for publicly available datasets
- A description of any restrictions on data availability
- For clinical datasets or third party data, please ensure that the statement adheres to our policy

Data supporting the findings of this work are available within the paper and its Supplementary Information files, or from the corresponding author upon request. Source data are provided with this paper.

Research involving human participants, their data, or biological material

Policy information about studies with human participants or human data. See also policy information about sex, gender (identity/presentation), and sexual orientation and race, ethnicity and racism.

Reporting on sex and gender	n/a
Reporting on race, ethnicity, or other socially relevant groupings	n/a
Population characteristics	n/a
Recruitment	n/a
Ethics oversight	n/a

Note that full information on the approval of the study protocol must also be provided in the manuscript.

Field-specific reporting

Please select the one below that is the best fit for your research. If you are not sure, read the appropriate sections before making your selection.

☒ Life sciences ☐ Behavioural & social sciences ☐ Ecological, evolutionary & environmental sciences

For a reference copy of the document with all sections, see nature.com/documents/nr-reporting-summary-flat.pdf

Life sciences study design

All studies must disclose on these points even when the disclosure is negative.

Sample size	Sample sizes were not predetermined, but chosen to be similar to common sample sizes in previous studies in the field.
Data exclusions	We did not exclude any data points arbitrarily.
Replication	All attempts at replication were successful. The number of replicates is indicated in the corresponding figure legend and/or in the corresponding material and method section.
Randomization	All samples from each experiments was collected randomly for further analysis (e.g. qRT-PCR and western blot assay).
Blinding	All investigation were blinded to group allocation during data collection and analysis.

Reporting for specific materials, systems and methods

We require information from authors about some types of materials, experimental systems and methods used in many studies. Here, indicate whether each material, system or method listed is relevant to your study. If you are not sure if a list item applies to your research, read the appropriate section before selecting a response.

Materials & experimental systems

n/a	Involved in the study	
☐	☒	Antibodies
☒	☐	Eukaryotic cell lines
☒	☐	Palaeontology and archaeology
☒	☐	Animals and other organisms
☒	☐	Clinical data
☒	☐	Dual use research of concern
☐	☒	Plants

Methods

n/a	Involved in the study	
☒	☐	ChIP-seq
☒	☐	Flow cytometry
☒	☐	MRI-based neuroimaging

Antibodies

Antibodies used

1.WYMV CP-specific antibody (prepared by Huaan Biotechnology Co., Ltd, Hangzhou, Zhejiang, China, and stored in our lab).
2. Flag, His, MBP, GST and GFP monoclonal antibody (TransGen Biotech, Beijing, China, HT201-01, HT501-01, HT701-01, HT601-01 and HT801-01).
3. RFP monoclonal antibody (Abbkine Scientific Co., Ltd., California, USA, ABM40169).
4. phospho-p44/p42-specific antibody (1:5000, Cell Signaling Technology, Massachusetts, USA, Cat. No. #4370).
5. HRP (Abbkine Scientific Co, California, USA, Cat. No. A21000).
6. phosphoserine-antibody (Sigma-Aldrich, Shanghai, China, Cat. No. SAB5200086).
7. H3 antibody (Cell Signaling Technology, Massachusetts, USA, Cat. No. #4499).
8. PEPC antibody and H+-ATPase antibody (Amyjet Scientific, Wuhan, China, Cat. No. AS09-458 and AS07-260).
9. Anti-Mouse and Rabbit (Abbkine Scientific Co., Ltd., California, USA, A21010 and A21020).

Validation

All antibodies expect for WYMV CP antibody are commercially available and validated in the literature as cited on the manufacturer's websites as well as by the datasheets they provide. Antibody validation and validation criteria are available on the following websites:
Flag antibody: https://www.transgen.com/antibody_tag/371.html
His antibody: https://www.transgen.com/antibody_tag/385.html
MBP antibody: https://www.transgen.com/antibody_tag/389.html
GST antibody: https://www.transgen.com/antibody_tag/388.html
GFP antibody: https://www.transgen.com/antibody_tag/390.html
RFP antibody: https://www.abbkine.com/product/rfp-tag-monoclonal-antibody-abm40169/
Phospho-p44/p42-specific antibody https://www.cellsignal.cn/products/primary-antibodies/phospho-p44-42-mapk-erk1-2-thr202-tyr204-d13-14-4e-xp-rabbit-mab/4370
H3 antibody:https://www.cellsignal.cn/products/primary-antibodies/histone-h3-d1h2-xp-rabbit-mab/4499
PEPC antibody: https://www.amyjet.com/products/AS09-458-HRP.shtml
H+-ATPase antibody: https://www.amyjet.com/products/AS07-260.shtml
Anti-Mouse : https://www.abbkine.com/product/hrp-goat-anti-mouse-igg-a21010/
Anti-Rabbit: https://www.abbkine.com/product/hrp-goat-anti-rabbit-igg-a21020/
HRP: https://www.abbkine.cn/?s_type=productsearch&s=A21000
CP-specific antibody was available and validated which confirmed in a previous study (Zhang et al., Nature Communications, 2022, 13: 6576)

Dual use research of concern

Policy information about dual use research of concern

Hazards

Could the accidental, deliberate or reckless misuse of agents or technologies generated in the work, or the application of information presented in the manuscript, pose a threat to:

No	Yes	
☒	☐	Public health
☒	☐	National security
☒	☐	Crops and/or livestock
☒	☐	Ecosystems
☒	☐	Any other significant area

Experiments of concern

Does the work involve any of these experiments of concern:

No	Yes	
☒	☐	Demonstrate how to render a vaccine ineffective
☒	☐	Confer resistance to therapeutically useful antibiotics or antiviral agents
☒	☐	Enhance the virulence of a pathogen or render a nonpathogen virulent
☒	☐	Increase transmissibility of a pathogen
☒	☐	Alter the host range of a pathogen
☒	☐	Enable evasion of diagnostic/detection modalities
☒	☐	Enable the weaponization of a biological agent or toxin
☒	☐	Any other potentially harmful combination of experiments and agents

Plants

Seed stocks	all seeds which used in this study was stored in our lab.
Novel plant genotypes	In this study, we obtained the TaRD21A transgentic over-expression lines and knock out TaRD21A in wheat plant. To produce transgenic wheat plants overexpressing TaRD21A, the full-length TaRD21A sequence from YM158(S) and Fielder(R) was cloned behind the ubiquitin promoter in a pUbi:00 vector to produce pUbi: TaRD21AS and pUbi: TaRD21AR. A pAHC20 vector carrying a selective bar gene was cotransformed together with pUbi: TaRD21AS or pUbi: TaRD21AR into immature embryos of YM158(S) by particle bombardment. Two positive line was obtained via PCR using ubiquitin promoter-specific primers. To knock out TaRD21A, sgRNAs specific for the three TaRD21A copies were designed. A sgRNA-clustered, regularly interspaced, short palindromic repeats (CRISPR)-CRISPR-associated 9 (Cas9) expression vector was used for transforming immature embryos of the wheat cultivar Fielder. One TaRD21A knockout line where the D-genome copy alone was affected (tard21ar-2D-KO) and another one knockout line where all three copies of mutant TaRD21A were disrupted (tard21ar-KO) via PCR/RE assay
Authentication	To determine the off-target gene editing, we sequenced a gene highly resembling TaRD21AR in tard21ar-KO and tard21ar-2D-KO, and confirmed that the editing construct was not target to the other genes.
